# Real-Time Monitoring of Oxygen-Consumption Rate in Mouse Liver Slices Incubated in Organ-on-a-Chip Devices

**DOI:** 10.1021/acs.analchem.4c00355

**Published:** 2024-09-30

**Authors:** Ruby E. H. Karsten, Konstanze Gier, Jean-Paul S. H. Mulder, Maciej Grajewski, Peter Olinga, Elisabeth Verpoorte

**Affiliations:** † Pharmaceutical Analysis (XB20), Groningen Research Institute of Pharmacy, University of Groningen, 9713 AV Groningen, The Netherlands; ‡ Pharmaceutical Technology and Biopharmacy, Groningen Research Institute of Pharmacy, University of Groningen, 9713 AV Groningen, The Netherlands

## Abstract

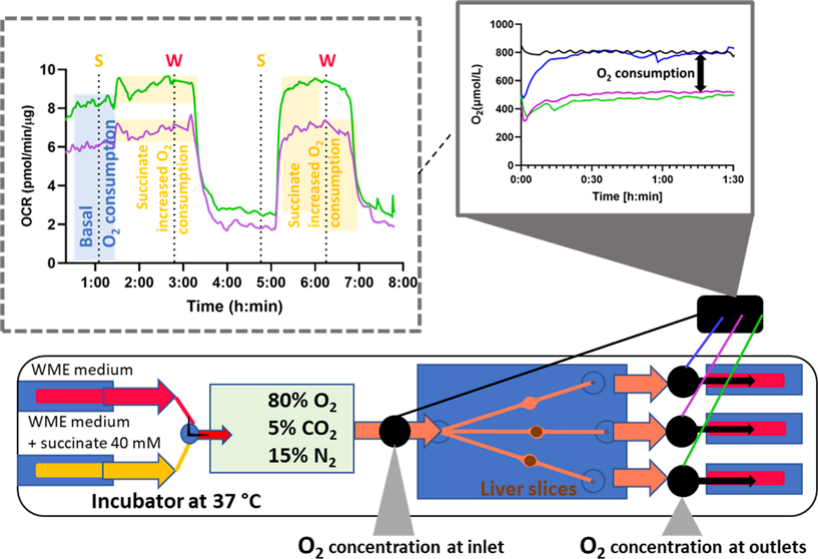

We developed an organ-on-a-chip (OOC) based on precision-cut liver slices to assess liver function in real-time, both in health and disease, in a controlled and noninvasive manner. We achieved this by integrating fiber-optic-based oxygen sensors before and after the microchamber in which a liver slice was incubated under flow, to measure oxygen concentrations in the medium in real time. We first demonstrated that the basal oxygen consumption rate (OCR) of liver slices is a reliable indicator of liver slice viability. By monitoring basal OCR (2.9–5.7 pmol O_2_/min/μg protein) in incubation medium, we found that it correlated well to cellular adenosine triphosphate (ATP) content (3.0–7.9 pmol/μg protein) (*r* = 0.82, *p* < 0.0001). Second, we induced a diseased state in liver slices by targeting the mitochondria, as they play a critical role in liver function and disease. We exposed the liver slices to succinate in abundance (40 mM) for short periods (1 h) to rapidly boost mitochondrial OCR. Two successive treatments of succinate increased the OCR of liver slices by 1.5 pmol/min/μg each time. However, between treatments, the liver slice OCR did not return to its basal OCR, instead decreasing drastically by 60–70%, suggesting succinate toxicity. We confirmed this with ATP analysis (1.0 pmol/μg protein) and hematoxylin and eosin staining, which showed tissue necrosis and apoptosis. Our system could be an advantageous model for future studies assessing liver (patho)physiology in response to potentially toxic drugs or lifestyle-related liver diseases.

To better understand the conditions under which the liver fails *in vivo*, it is important to truly recapitulate liver physiology *in vitro*. Since it has become more evident that the study of complex biological processes requires complex biological models, various liver tissue models have been developed. These *in vitro* models allow induction of the mechanisms that cause liver diseases, in order to study the liver’s physiology as it malfunctions and to investigate ways to reverse these processes. Precision-cut liver slices are one such organotypic model that can be manipulated under pathological conditions to recapitulate metabolic-associated fatty liver disease, fibrosis, and cholestasis.^1−3^ Slices consist of intact liver tissue, with extracellular matrices, multiple cell types, and cellular polarity. The well plate liver-slice model has proven to be a good one for the study of pathological processes in the liver.^4−6^ However, if the experimental aim is to achieve precise control over the microenvironment of the tissue, this may be better accomplished by introducing the tissue slice to a microfluidic flow system, to realize an organ-on-a-chip (OOC).^7,8^ OOCs have gained interest over the past decade because they offer a route to more advanced *in vitro* studies of (engineered) organ tissue. They capture the structural, mechanical, chemical, and communicative complexities of *in vivo* systems,^8^ such as all-important blood flow. Key for the microfluidic configuration of an OOC are considerations like the removal of waste products and metabolites, and proper distribution of oxygen and nutrients.

The design of OOCs also takes into account the *in situ* and in-line integration of sensors to characterize tissue response in real time. One of the most important parameters in the operation of an OOC for which sensors can be used is the monitoring of oxygen and its consumption during tissue incubation. A number of studies have successfully implemented oxygen sensors in liver-on-a-chip systems and shown the additional value of real-time oxygen measurements during tissue response.^9−11^ Oxygen sensing provides precise information about the oxygen supply and distribution in the incubation setup and allows insight into setup stability during experiments. Besides improving the incubation setup, measurements of cellular oxygen consumption also yield information about cellular viability and indicate the metabolic activity of the cell.^9,12^ The major pathway (>90%) of cellular oxygen consumption is mitochondrial respiration, in which the electron transport chain in the mitochondria utilizes oxygen to produce the main energy source of the cell, adenosine triphosphate (ATP). As oxygen is inherently involved in the production of ATP, it can be used as an indicator of many cellular processes. Thus, when investigating the (patho)physiology of the liver by monitoring tissue response to external and internal factors, real-time oxygen consumption by the tissue has great added value. Current liver-on-a-chip models^9−11^ with oxygen-sensing capability mainly focus on the use of primary hepatocytes rather than engineering more complex tissue. As a result, nonparenchymal cells for cell–cell interaction are generally omitted.^9,11,13−15^

In this study, our goal was to develop an OOC based on organotypic liver slices, with integrated oxygen sensors to assess liver function in health and disease in real time, in a controlled and noninvasive manner. When designing a microfluidic chip to include real-time oxygen measurement, choosing the right oxygen-sensing system and chip material is dependent on the application. In this study, optical sensors have been selected because they can be used noninvasively and are highly suitable for continuous monitoring.^16,17^ Optical oxygen sensors used in OOCs include polystyrene microbeads (Ø 50 μm) loaded with ruthenium-phenanthroline-based phosphorescence dye^9,11^ or sensor foils covering the incubation chamber.^18^ While these are good options, we believe that real-time oxygen measurements in microfluidics would benefit from quantitative sensors placed locally in the OOC setup, rather than foils or beads. This is particularly true in this case where the total oxygen consumption of liver slices is of interest, as was previously demonstrated by Oomen in our lab.^19^

This earlier work focused on the design and implementation of a 6-chamber polycarbonate (PC) device for individual incubation of 6 slices. Each slice was perifused by medium entering a 75-μL chamber from underneath and exiting the chamber from above, much like the device concept described by van Midwoud et al.^20^ Oomen observed a strong dependence of slice viability and morphology on flow rate, and oxygen consumption rate measurements for precision-cut liver slices were presented for the first time (rat slices were used).^19^

The work presented in this paper builds on this earlier study, though our device differs significantly from the 6-chamber device described above. We have developed a chip with a single inlet for three incubation chambers, two of which are for duplicate incubation of liver slices, and one empty control chamber to account for any procedural changes. Notably, liver slices (from mouse in our case) are suspended in a chamber that is perfused over its lower surface with medium introduced from a channel in the horizontal plane of the device (Figure 1). Our single-inlet device allows for a simplified incubation setup, in which it is possible to easily switch between different media and thus configure drug dosing studies. As in the previous study, fiber-optic-based oxygen sensors located before the inlet and at the chamber outlets of the chip provided for real-time oxygen measurements in the medium,^19^ resulting in highly quantitative oxygen consumption-rate data for liver tissue.

**Figure 1:**
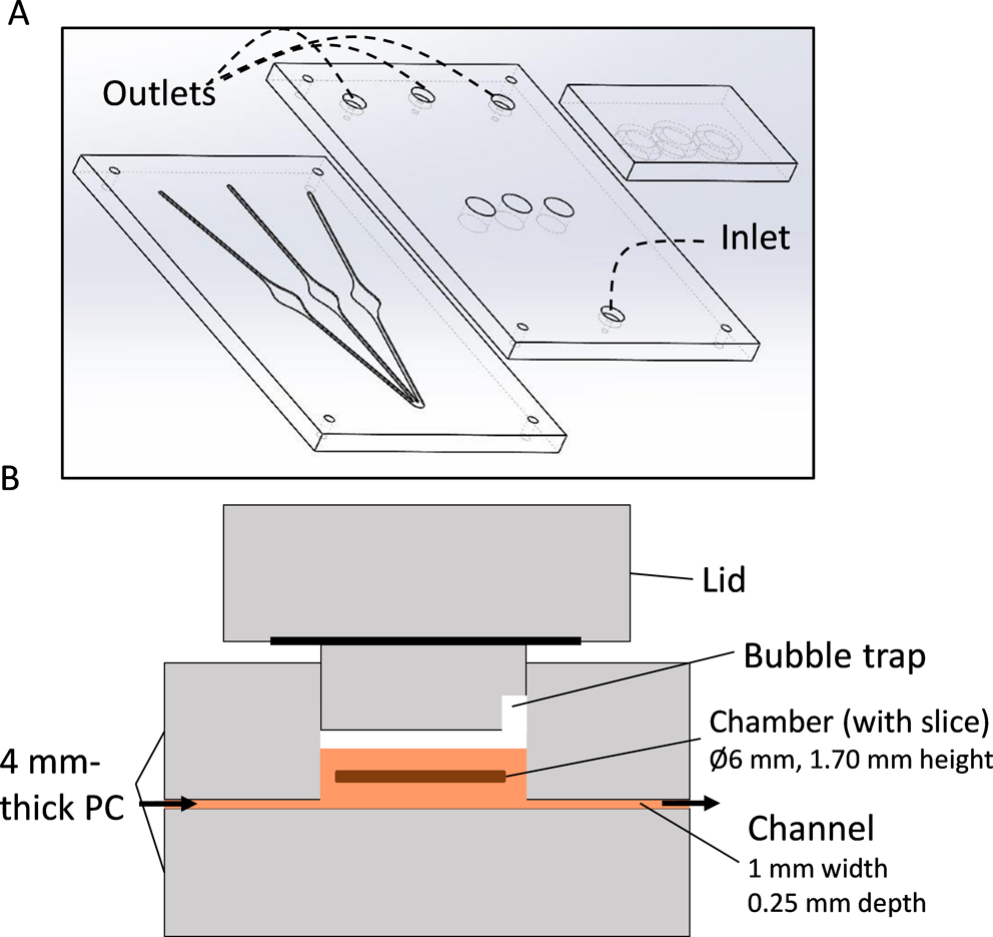
Assembly of the second-generation incubation chip. (A) Exploded view of the chip. Three 4-mm-thick PC pieces (gray) were prepared and assembled. From left to right: Bottom part, middle part, and top part (lid). (B) Cross-section of the device containing medium (orange), and a liver slice (brown). Bottom and middle parts were bonded to one another with a laser welding technique (Memetis GmbH, Germany). The bottom part contains the channels (1.00 mm width, 0.25 mm depth) and the lower section of the chambers (Ø 6.00 mm, 0.25 mm depth). The middle part seals the channels and contains holes for the inlet (recess depth: 1.50 mm; recess diameter: Ø 4.75 mm; central hole diameter: Ø 1.30 mm), outlets (same dimensions as inlet), and incubation chambers (Ø 6.00 mm). The upper part (lid) reversibly closes the chip by applying pressure on the O-rings (black). The lid includes a cylindrical protrusion which, when inserted into the chamber, renders a chamber volume of 48 μL. The protrusion of the lid has a small gap that functions as a bubble trap. Medium flow direction is indicated with arrows. The figure is not to scale.

The choice of chip material also depends on the specific application. While poly(dimethylsiloxane) (PDMS) is commonly used in microfluidics, its gas permeability poses challenges for quantitative analysis of oxygen consumption by liver slices. This is because the PDMS device is constantly at equilibrium with oxygen in the surroundings, and serves as an oxygen reservoir to replenish oxygen consumed by a liver slice in the device. Thus, an oxygen-impermeable material like PC is preferable. PC offers transparency and stability, ensuring controlled environments for accurate analysis without external oxygen influences.

In this study, we have developed a gas-impermeable microfluidic device micromilled in PC for the incubation of mouse precision-cut liver slices in a highly controlled fashion. We supplied the chip with 80% oxygen-saturated medium using a diffusion-based oxygenation module previously described by Oomen.^19^ Our first aim was to correlate the basal oxygen consumption rate (OCR) of a slice to its ATP content, measured after homogenization. In this way, we could determine whether basal OCR is a noninvasive proxy for ATP when monitoring tissue viability, which has not been done previously by us or others. We hypothesize that high basal OCRs should correspond to high levels of intracellular ATP. Second, we induced a diseased state in liver slices by targeting mitochondria with succinate, as mitochondria play a critical role in liver function and disease. Succinate is a substrate of the TCA cycle and provides one of the electrons to the electron transport chain,^21^ leading to enhanced ATP synthesis and thus oxygen consumption in the mitochondria when supplemented in medium.^22^ This phenomenon has been previously observed in human and rat liver slices.^19,23^ With this experiment, we aimed to demonstrate our system’s capability to detect and monitor fluctuations in mitochondrial oxygen consumption by quantifying liver-slice oxygen consumption. To do so, we periodically added significant amounts of succinate (40 mM) to the medium for short periods (1 h) to rapidly boost oxygen consumption in liver slices and measure it in our system. Additionally, we measured the basal oxygen consumption in the recovery periods (1 h) between succinate treatments to observe possible toxicity of succinate. Lastly, we assessed the viability of the tissue after experiments by means of a cellular ATP content assay and hematoxylin and eosin (H&E) staining, to correlate this to the OCR of liver slices during experiments.

## Materials and Methods

### Animals

Male C57BL/6J mice aged 8 to 11 weeks were collected from the University Medical Center Groningen’s Central Animal Facility. Mice were kept in a temperature- and humidity-controlled room with a 12-h light/dark cycle, with food and water ad libitum.

The experiments were authorized by the University of Groningen’s Animal Ethical Committee (CCD number AVD105002017884) and carried out in compliance with EU Directive 2010/63/EU on animal experiments.

### Preparation and Incubation of Mouse Liver Slices

Precision-cut mouse liver slices (5 mm in diameter, 250 μm thick) were prepared and incubated at 37 °C in an atmosphere of 80% O_2_, 15% N_2_, and 5% CO_2_ in well plates in Williams Medium E supplemented with glucose and gentamycin (WMEGG) according to protocols outlined previously.^5,24^ After 0, 24, or 48 h of incubation in the well plates, slices were inserted into the microfluidic device. See the Supporting Information (SI), Section 2.1 (S2.1) for a more detailed description. The protocol for incubation of liver slices in the chip is explained under “Real-time oxygen measurements” (*vide infra*).

### ATP and Protein Analysis

Protocols for measuring ATP and protein content in liver slices have been previously described;^5,24^ a more detailed description of these protocols can be found in the SI, S2.2. The ATP values were normalized to the total protein content of the slice to correct for the size of the slice, as were OCR; this procedure is further described under ‘Real-time oxygen measurements’ (*vide infra*). The normalized ATP values (pmol/μg protein) were then correlated to the mean OCR (pmol O_2_/min/μg protein) of the same liver slice.

### Hematoxylin and Eosin (H&E) Staining

After incubation in the microfluidic platform, liver slices were fixed for 24 h in 4% formaldehyde in phosphate-buffered saline (PBS) solution at 4 °C, and stored in 70% ethanol at 4 °C until analysis. A pathologist not familiar with the study evaluated slice morphologies to assess tissue viability as a function of the amount of necrotic tissue in a slice. See the SI, S2.3, for a more detailed description of the method.

### Chip Design and Fabrication

Two generations of our liver slice incubation device were developed in this study; the second-generation device is shown in Figure 1. The two devices have a lot of similarities, as they have the same internal chip structures and dimensions (technical drawings with all dimensions are given in the SI, S2.4, Figures S1–S5; assembly of the first-generation of the incubation chip is shown in Figure S6). The chip structure consists of three incubation chambers with a common inlet and three separate outlets (Figure 1). The second-generation device consisted of three 4 mm-thick PC layers (Figure 1A). The structures in the individual parts were prepared by micromilling. The lowest PC layer in both devices contains the microfluidic channels and incubation chambers (Figure 1A). Channels have a width of 1.00 mm and depth of 0.25 mm, while slice chambers have a diameter of 6.00 mm and depth of 0.25 mm (Figure 1B). The middle PC layer closes the microfluidic channels off, and contains four small cylindrical holes (Ø 1.30 mm) to serve as the sole microfluidic channel inlet and three outlets (Figure 1A). The four through-holes are centered in larger circular recesses, the dimensions of which are given for both device generations in the SI, S2.4 (Figures S1–S5). This layer also embeds a row of three larger cylindrical holes (Ø 6.00 mm, *h* = 1.00 or 4.00 mm), where h is equal to the thickness of the PC layer used to create accessible slice incubation chambers (Figure 1A).

The second-generation device differs from the first-generation device with respect to assembly, as we noted that first-generation devices exhibited some oxygen leakage and were not that durable. This was because the different layers in this device were bonded together with biocompatible adhesive tape (3 M, Bracknell, U.K.), which did not retain its adhesive properties for more than a set of experiments. In contrast, the second-generation incubation device used laser welding to bond the lower and middle PC parts (Memetis GmbH, Karlsruhe, Germany). This improved chip durability and oxygen impermeability.

**Figure 2:**
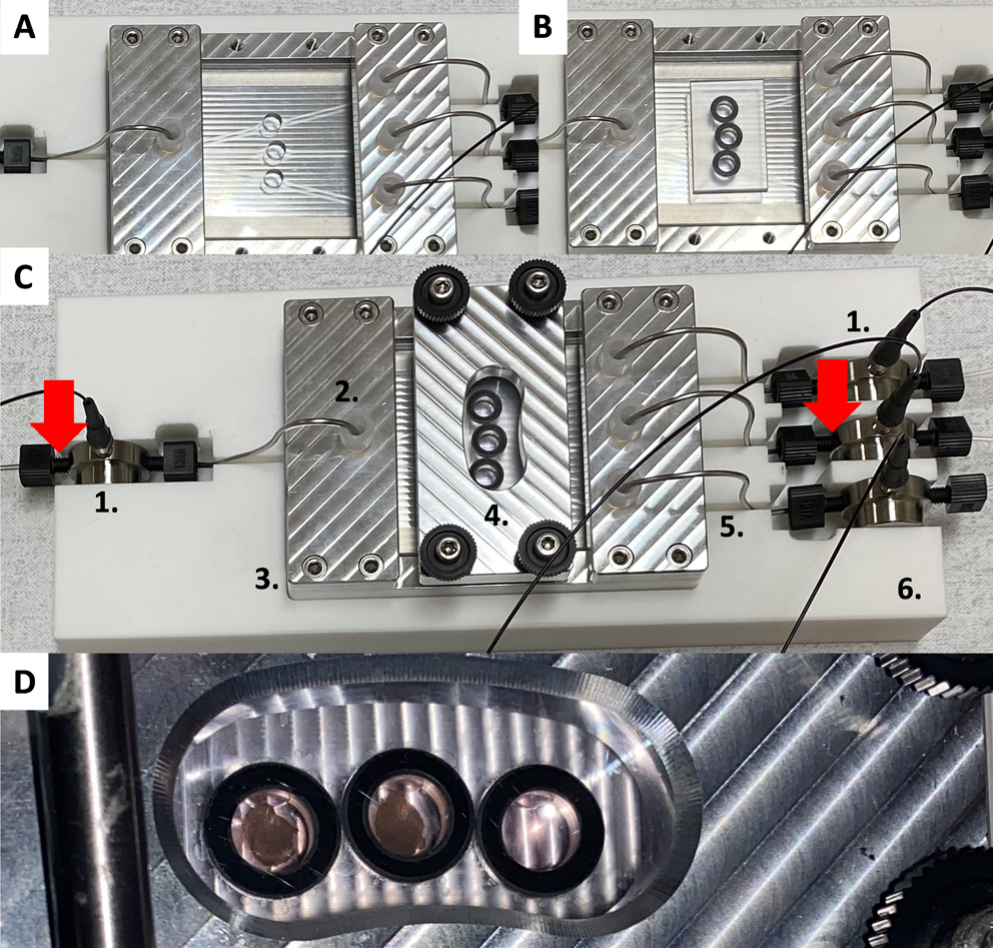
Assembly of the second-generation device in the chip holder. Incubation setup: (A) Lower part of the holder that the chip is placed in. The chip is slid into the holder by mounting two side plates to hold the device in place. The side plate on the left-hand side contains holes through which stainless steel tubing connects the device to the medium at the inlet. On the right-hand side, the side plate contains three holes to accommodate the stainless steel tubing that transports medium from the device. (B) Sealed chip with the lid. The O-rings (black) placed around the cylindrical protrusions that close the three slice chambers are clearly visible, due to the transparency of PC. (C) Complete incubation setup: 1. Four optical flow-through oxygen sensors, with temperature sensors on the exterior (red arrows) connected to the inlet and middle outlet. 2. Stainless steel tubing through the holder connecting to the inlet of the chip. 3. Metal holder for the chip. 4. Metal cover to seal the lid to the device. 5. Stainless steel tubing through the holder’s slide plates connecting to the outlets of the chip to accommodate transport of medium from the device to the oxygen flow-through sensors. 6. 3D-printed case housing the holder of the chip and the oxygen sensors. (D) Closed chip with two mouse precision-cut liver slices incubated in the middle and left chamber.

To ensure that there were no bubbles formed in the incubation chambers when closing, the chambers were completely filled with medium and a few drops of medium were added to the cylindrical structures of the lid. Additionally, the dimensions of the protruding cylindrical structures of the lid ensured a small gap between these and the medium to further ensure bubble-free closing of the device. The inlet and outlets of the second-generation chip were connected to stainless-steel tubing (3.2 mm OD, 0.8 mm ID, BGB Analytik, Harderwijk, The Netherlands) by means of finger-tight flat fittings (BGB Analytik, Harderwijk, The Netherlands). These fittings were fixed in threaded holes in the stainless steel holder to hold the tubing in place (Figure 2).

The other ends of the inlet and outlet stainless-steel tubing were then connected with finger-tight cone fittings (BGB Analytic, Harderwijk, The Netherlands) (Figure 2C) to four in-line integrated, optical flow-through oxygen sensors (internal volume of 2.1 μL) (FTCM- PSt7–02, Presens, Regensburg, Germany). These connections were oxygen-impermeable and leakage-proof, and made it possible to connect and disconnect the device easily from the peripheral experimental system, which was not possible in the first-generation device. Lastly, two temperature sensors (PT100, Presens, Regensburg Germany) were installed to measure the temperature more precisely at the exterior of the oxygen sensors, positioned at the inlet and the middle outlet of the chip. These sensors provided constant feedback to the software calculating the oxygen concentrations in the medium from sensor readouts (Figure 2C).

### Set-up

**Figure 3:**
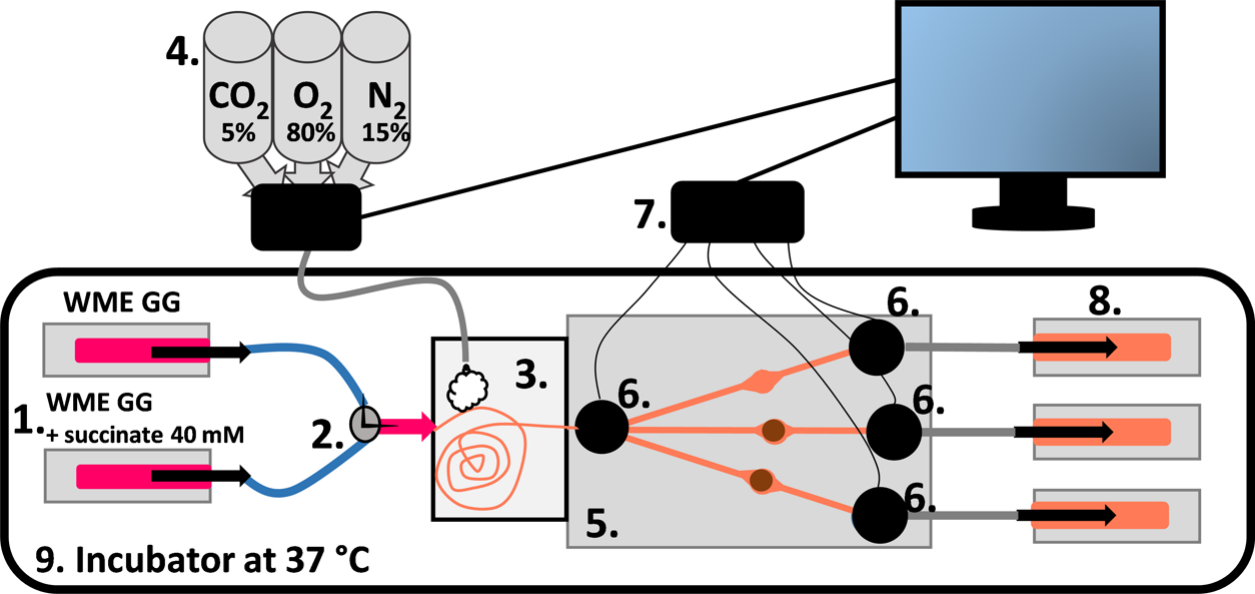
Schematic depiction of the complete precision-cut liver slice incubation setup. Two 20-mL medium-filled syringes were clamped onto a syringe pump set to 60 μL/min (1) and attached to a three-way valve (2) to switch between supplemented Williams’ medium E (WMEGG) and succinate-supplemented WMEGG (1). The medium was oxygenated as it was pumped through silicone tubing coiled in a diffusion-based oxygenation box (3) containing a gas mixture (80% O_2_, 15% N_2_, 5% CO_2_) (4) before entering the chip (5). Liver slices were incubated in two out of three incubation chambers, and one incubation chamber was used as a control (5). Oxygen sensors were placed at the inlet and outlets of the chip (6) and oxygen measurements were recorded on the computer outside the incubator (7). Three pumps were installed at the outlets to aspirate equal flow rates of 20 μL/min (8). The black frame marks the incubator set to 37 °C in which the setup was placed (9).

The entire setup for operating both generations of OOCs is schematically depicted in Figure 3. Two 20 mL, medium-filled syringes were clamped onto a syringe pump (NE-1000 Programmable Single Syringe Pump, KF Technology, Roma, Italy) set to 60 μL/min. These syringes were connected to Teflon tubing (0.75 mm OD, 0.6 mm ID, 25 cm long; Polyfluor, Breda, The Netherlands) by needles with Luer-lock connections (0.8 mm OD, 0.6 mm ID, 40 mm long; BD Biosciences, San Jose, California). The tubing was in turn attached to a three-way valve to switch between WMEGG and succinate-supplemented WMEGG. The medium was oxygenated before entering the chip as it was pumped through three meters of silicone tubing (OD 0.8 mm, ID 0.5 mm; Fisherbrand, Loughborough, U.K.) coiled in a box containing a gas mixture (80% O_2_, 15% N_2_, 5% CO_2_). Diffusion of gases over the wall of the silicone tubing ensured equilibrium of the medium with the gas in 9.8 min (residence time of medium in the tubing at a flow rate of 60 μL/min).^19^ A mass-flow sensing system consisting of three mass-flow controllers connected to a common mixing chamber (Flow-SMS, Bronkhorst High-Tech B.V., Ruurlo, The Netherlands) was used to fill the diffusion-based oxygenation box with the gas mixture. Additionally, some wet tissue paper was placed in the oxygenation box to maintain humidity. Liver slices were incubated in two out of three incubation chambers, and one incubation chamber was used as a control. Flow-through oxygen sensors were installed in-line, one before the inlet and one each after each outlet (four sensors in total). These sensors were connected to a multichannel oxygen meter (OXY-4ST (G2)) (Presens, Regensburg, Germany), to record measured oxygen levels in the medium in terms of oxygen percentage during experiments. The actual oxygen sensor is located at the tip of an optical fiber fitted into the middle port of a circular, stainless-steel tee piece, with its sensing end positioned in the flow. The sensing principle is based on a coating containing a ruthenium dye. Fluorescence emitted by the dye is quenched by oxygen, with the degree of quenching being directly proportional to the amount of oxygen present in the medium. Three pumps were installed at the outlets and set to aspirate a flow rate of 20 μL/min to ensure equal distribution of the inlet flow of medium (60 μL/min) over all three incubation chambers. A flow rate of 20 μL/min was selected based on the liver-slice incubation study conducted by van Midwoud et al., in which slices were studied in a microfabricated 25-μL incubation chamber under a constant flow of medium at a flow rate of 10 μL/min.^20^ The resulting refreshment of medium in the chamber every 2.5 min was sufficient to ensure a constant and adequate supply of oxygen and nutrients to the liver slice to maintain metabolic activity and viability.^20^ To achieve a refreshment rate of approximately 2.5 min in our 48-μL incubation chamber, we selected a flow rate of 20 μL/min. We confirmed laminar flow in our device by calculating a Reynolds number of 0.12 (SI, S2.5), and determined the Péclet number for oxygen and glucose to be 26.7 and 90.3, respectively. These values indicate that advection (flow) significantly contributes to mass transport, with diffusion playing a lesser role.

The diffusion-based oxygenation box, medium pumping system, and chip were installed inside a custom-made transparent incubation cabinet constructed in PC. This incubation cabinet had integrated temperature control that was set to 37 °C (temperature controller Jumo iTRON PID Temperature Controller, 48 × 24 (1/32 DIN) mm 1 (Analogue), RS Components B.V., Haarlem, The Netherlands). The temperature sensors located in the exterior housing of the oxygen sensors provided feedback to the oxygen sensing software to accurately calculate the oxygen concentration in the medium at any given temperature. During experiments, the temperature ranged between 33 and 37 °C, due to opening and closing of the incubator when changing medium.

### Real-Time Oxygen Measurements

Before chip experiments, all four oxygen sensors were calibrated either individually or together using a 2-point calibration in the microfluidic setup (SI S2.6). Liver slices placed inside the incubation chambers of the chip were perfused with gas-equilibrated, heated WMEGG medium (80% O_2_, 5% CO_2_, 15% N_2_, 37 °C), with a flow rate of 20 μL/min per chamber (Figure 3). Liver slices were incubated under flow for 8 h, after which they were removed and tested for viability with an ATP assay or by H&E staining. The OCR of each liver slice was then compared to the ATP content or the morphology of the same liver slice, after correcting for the size of the slice by protein content. As a control, liver slices were incubated in shaken well plates containing WMEGG in an incubator for the same duration and under the same temperature and gas conditions as the OOC experiments. The schematic diagram describing the experimental design for the OOC experiments can be found in the SI S2.7, Figure S7.

During experiments, oxygen percentage was measured once a minute at the inlet at a flow rate of 60 μL/min. At the outlets, oxygen percentages were registered every minute at a flow rate of 20 μL/min. The data were then analyzed and converted to oxygen concentration expressed in pmol/μL based on ambient temperature and pressure, using Studio 2 software (Presens, Regensburg, Germany). The formula for the OCR of a slice is written in eq 1, where Coxy refers to oxygen concentration. The derivation of this formula can be found in the SI, S2.8. Note that oxygen concentrations were normalized according to the protein content of the slice studied; this parameter is included in eq 1. Equation 1 shows that the measured OCR is actually independent of Coxy at the device inlet, under the condition that Coxy at the outlets ≠ 0.
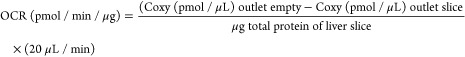
1

### Boosting Oxygen Consumption with Succinate

The same experimental conditions were used as described under “real-time oxygen measurements”, only in this experiment, the mouse liver slices were alternately incubated with WMEGG supplemented with and without sodium succinate dibasic hexahydrate (40 mM) (Figure 3) (Sigma-Aldrich, the Netherlands). The succinate-treated liver slices incubated in the chip for 8 h were then compared to untreated liver slices incubated in the well plates for the same duration. The liver slices were collected and processed further as described under “Real-time oxygen measurements” (*vide supra*).

### Statistical Analysis

A minimum of three mouse livers were used for each experiment, using liver slices in duplicate or triplicate from each liver. As the number of livers varied with each experiment, this information is given in the captions for figures presenting experimental data. GraphPad Prism 8.4.3 was used to analyze data statistically using a Student’s unpaired two-tailed *t* test to compare two means or a one-way analysis of variance (ANOVA) to compare multiple means. One-way ANOVA was followed by either Tukey’s multiple comparisons test to compare all means within a data set or Dunnett’s multiple comparisons test to compare all means with their control mean. GraphPad Prism 8.4.3 was also used to perform the correlation analysis between OCR and ATP content. Differences between groups were considered significant when *p* < 0.05.

## Results and Discussion

### System Development and Characterization

We developed a protocol for calibration of the oxygen sensors and characterized both generations of OOC. This involved determining the delay time of the system, meaning the time needed for the oxygen-enriched medium to reach the sensors of the inlet and outlets. The equilibration time of the system, namely the time for the oxygen concentration to stabilize within the system, was also determined. This information can be found in the SI, S3.1.

**Figure 4:**
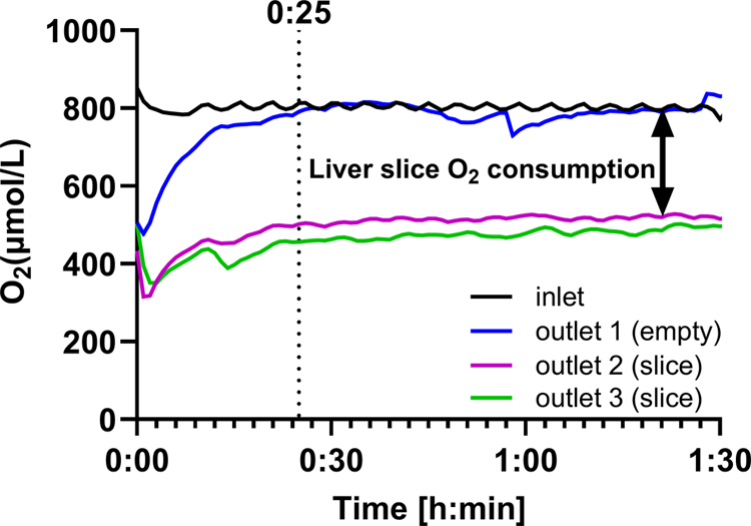
System run of the second-generation device with Williams medium E supplemented with glucose and gentamycin (WMEGG) with 800 μmol/L dissolved oxygen at 37 °C for 1.5 h. The WMEGG was exposed to 80% oxygen in the diffusion-based oxygenation box. The perfusion rate of the inlet is 60 μL/min, which is divided over three outlets, each perfused at a rate of 20 μL/min. Liver slices were incubated in chambers connected to outlet sensors 2 and 3. Outlet 1 was connected to an empty incubation chamber and used as a reference to calculate the OCR of the liver slices (indicated with a black arrow). The outlet sensor 1 (no slice present) was equilibrated 25 min after closing the device. The graph for the first-generation device can be found in the SI, S3.1, Figure S10.

Experiments with liver slices (Figure 4) were performed at 37 °C with 80% O_2_, 5% CO_2_, and 15% N_2_, as this was previously shown to be optimal for liver slice incubation.^25,26^ An exemplary graph of the oxygen measurements in a second-generation device during liver slice incubation is shown in Figure 4 (the graph for the first-generation device can be found in the SI, S3.1, Figure S10). Before performing experiments, the system was fully equilibrated with medium at 80% oxygen. Liver slices were placed in chambers 2 and 3, which were connected to outlet sensors 2 and 3, respectively. One empty chamber was connected to outlet sensor 1; this chamber was used as a control for the nonoxygen consumption-related oxygen loss between the inlet and outlets in the first-generation device. Outlet 1, corresponding to a chamber containing no slice, was used as a reference to calculate the OCR of liver slices in both device generations (eq 1) (Figure 4). In the second-generation device, however, the reading at the outlet sensor of the reference chamber is about the same as at the inlet sensor (Figure 4), as oxygen loss within the device had been minimized to essentially zero. This way, the third incubation chamber can also be used for slice incubation, providing the results in triplicate instead of duplicate.

After inserting the liver slices to start an experiment, outlet sensor 1 in the absence of a liver slice reached a stable oxygen concentration after 19.5 ± 6.2 min (*n* = 12 experiments for both device generations). Data for an experiment in the second-generation device is shown in Figure 4. Both device generations had equal equilibration times. As a result of liver-slice oxygen consumption, outlet sensors 2 and 3 showed lower oxygen concentrations than outlet sensor 1, namely 282 and 312 μmol/L, respectively, for the liver slices shown in Figure 4. To establish the OCR of the liver slices, the oxygen consumption per liver slice was then normalized for μg protein in a slice per minute using eq 1. Importantly, liver slice behavior was the same in both devices as will be explained under “Basal OCR of liver slices” (*vide infra*). Therefore, the data presented in the rest of the paper does not distinguish between the two device designs.

### Real-Time Monitoring of Oxygen-Dependent Metabolism in Murine Liver Slices

#### Viability Assessment of Liver Slices after Incubation in the Chip and Well Plate

Our investigation into liver slice viability within the liver-on-a-chip system and traditional well plates revealed comparable levels of viability as assessed by H&E staining over an 8-h period. Both systems showed approximately 40–60% viable cells, as scored by an independent pathologist. Differences in ATP content between the two incubation methods were observed, with a significant decrease noted in slices under continuous flow (4.7 pmol/μg), compared to those under dynamic shaking (7.9 pmol/μg). Detailed H&E and ATP data can be found in the SI (S3.2, Figure S11).

The lower ATP content in liver slices incubated under continuous flow is reproducible and furthermore consistent with previous work,^19^ though the reasons for this remain unclear. Despite the reduction in ATP, previous studies have demonstrated consistent viability and function of liver slices in chip systems compared to well plates.^20,27,28^ This reproducible perfused-slice behavior, combined with the observed viability of 40–60% hepatocytes in both the chip and well-plate systems, was deemed sufficient for our experimental objectives with respect to assessment of liver-slice OCR.

#### Basal OCR of Liver Slices

**Figure 5:**
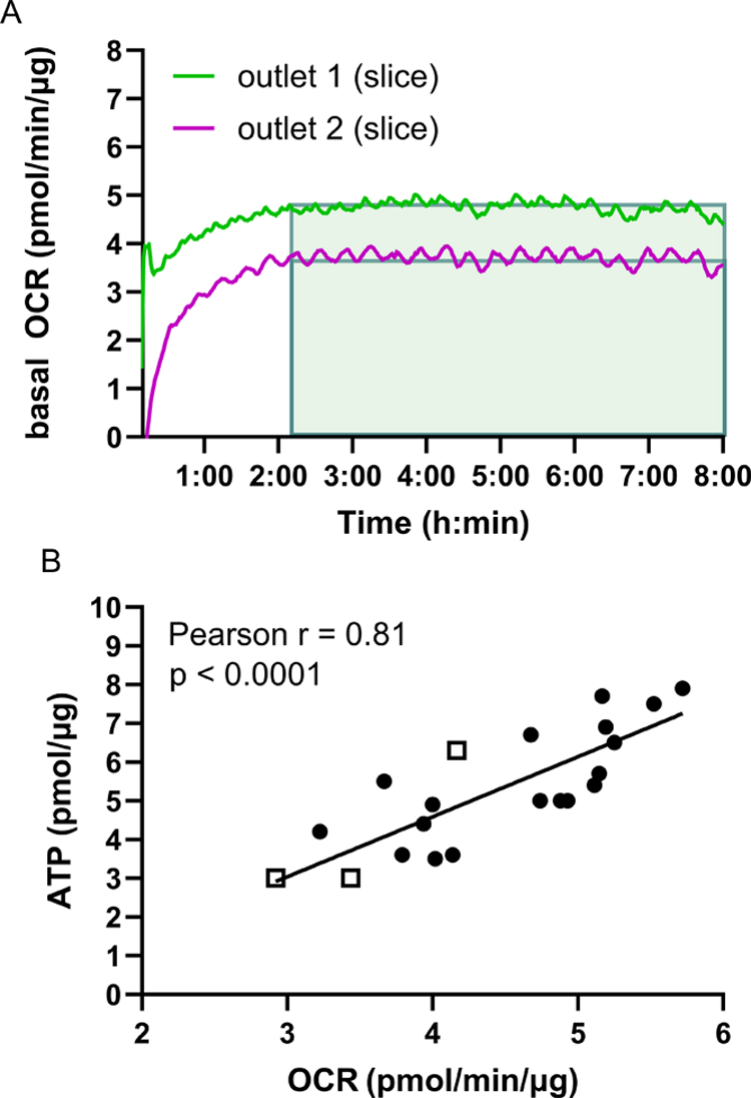
Mean OCR of liver slices strongly correlates to intracellular ATP content. (A) OCR (pmol/min/μg) of two liver slices incubated for 24 h in a well plate followed by 8 h of incubation in the chip, at a flow rate of 20 μL/min. (B) Correlation of mean basal OCR (pmol/min/μg) of liver slices and ATP content (pmol/μg) of the same liver slice. *N* = 6 mice, 2–4 liver slices per mouse for the first device (solid black circles), and *N* = 3 mice, 1 liver slice per mouse for the second device (open squares). Liver slices were incubated for 1, 24, or 48 h in well plates before 8 h of incubation in the chip. Individual data points of (B) before and after correction for protein are shown in the SI, S3.3, Table S1. Pearson’s correlation analysis was performed.

The basal OCR of liver slices was assessed for viability by measuring OCR in slices from 8 mice over 8 h (Figure 5A) and correlating it with intracellular ATP content (Figure 5B). Mouse liver slice OCR ranged from 2.9 to 5.7 pmol/min/μg, similar to rat liver slices incubated under the same conditions (between 2.8 and 5.3 pmol/min/μg).^19^ When rat liver slices were incubated at lower oxygen concentrations (0.3 pmol/min/μg at 21% oxygen), lower OCR were observed.^29^ Oxygen consumption varies not only due to the oxygen levels used for an experiment,^19,29^ but also because oxygen consumption of cells varies a lot between species, *e.g.*, rat liver slices have higher OCR than human liver slices,^23^ and mouse primary hepatocytes, more than human hepatic cell lines.^11^ Additionally, within species, the oxygen consumption varies depending on cell types^9,10^ and days of culture.^11^ This emphasizes the need to establish basal OCR for each model.

A strong correlation between mean ATP and OCR in mouse liver slices was observed, in which a high OCR corresponds to ATP-rich, viable hepatocytes (*p* < 0.0001, Pearson’s *r* = 0.81). This is an interesting result, as it means that we can use OCR measured in our system as a marker for slice viability (Figure 5B). Basal OCR above 4.5 pmol/min/μg correlated with an ATP level of about 5 pmol/μg, representing approximately 50% viable hepatocytes.^1^ The OCR and ATP values in Figure 5B were mostly obtained in the first-generation device (solid black circles). There were initial concerns that oxygen leakage from the first-generation device resulted in too little oxygen being available to satisfy liver slice metabolic demand.

However, studies have indicated that oxygen levels greater than 40% are more than sufficient to maintain liver slice viability and function.^30^ As shown in Figure S9, oxygen levels at the outlets of the chambers in the first-generation device were on the order of 54% for an inlet concentration of 80%, and therefore sufficient for adequate slice incubation. The results in Figure 5B corroborate this hypothesis, as OCR measurements in the second-generation, hermetically sealed device (open squares) using medium with an excess of oxygen (80%) fall within the same range as data obtained with first-generation devices. Previous findings reported weak correlation^31^ or no correlation at all^32^ between OCR and cellular ATP. This is due to the fact that any changes in environmental parameters like temperature or medium can change the amount of ATP generated per molecule of oxygen consumed by mitochondria.^33^ In contrast, our study reveals a strong correlation between OCR and ATP content, most likely due to the excellent control we can exert on tissue environment in our slice incubation system. Future research with our system could easily establish what oxygen percentage achieves optimal oxygen consumption for maximal viability in liver slices from mice or other species.

#### Boosting the OCR of Liver Slices under Flow by Succinate Treatment

In order to show that we can measure changes in OCR with our device, we induced a dynamic respiration process in our system by treating liver slices with succinate to selectively boost mitochondrial oxygen consumption. We chose succinate because it is a substrate in the electron transport chain targeting mitochondria specifically.^21^ Succinate has been shown to increase oxygen consumption in liver slices.^19,23,34,35^ These studies demonstrated that oxygen consumption in human and rat liver slices was significantly increased by a factor of 2 with the addition of 20 mM of succinate. Building on the study by Oomen,^19^ we performed a dosing experiment with high concentrations of succinate (1 h, 40 mM) to monitor the OCR response during succinate treatment and recovery (1 h) in real time. We introduced recovery periods without succinate, to observe the effect of subsequent bursts of mitochondrial oxygen consumption on basal OCR and whether these correlated to end-point viability assays.

**Figure 6:**
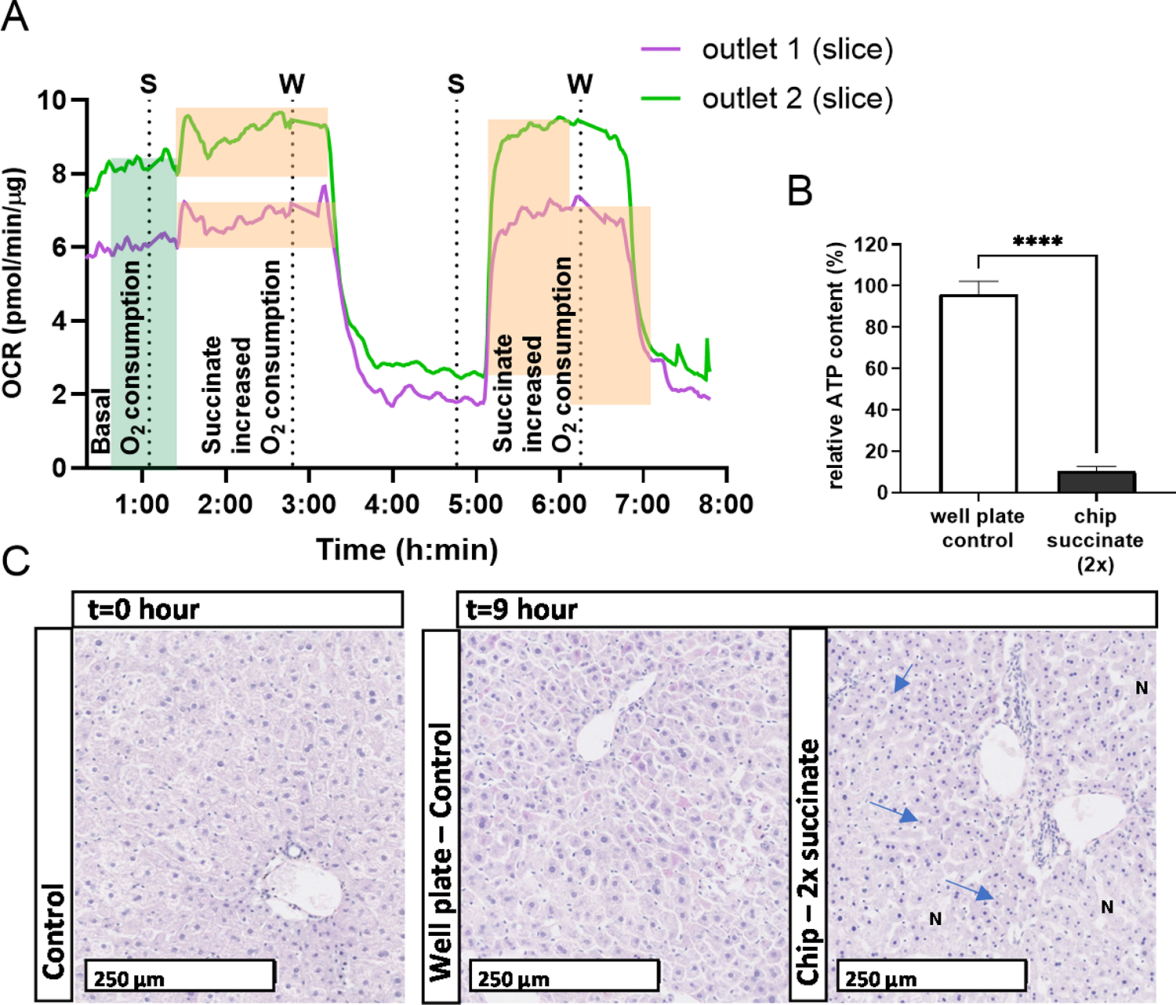
Rapid changes in OCR of liver slices in response to succinate treatment. (A) OCR (pmol/min/μg) of two liver slices incubated for 1 h in a well plate followed by 8 h in the chip, at a flow rate of 20 μL/min, with regular Williams medium E supplemented with glucose and gentamycin (W), and treated twice with succinate (40 mM) (S). (B) Corresponding relative ATP content (%) ± SEM of liver slices at 8 h treated twice (2×) with succinate compared to untreated liver slices in freshly filled well plates (control) (C) Corresponding hematoxylin and eosin staining of liver slices treated twice with succinate compared to untreated liver slices in well plates (control). Scoring of the number of viable hepatocytes showed decreased viability after two succinate treatments (9 h) compared to 0 h and the untreated liver slices (9 h), showing more necrosis (N) and pyknotic nuclei (arrows) indicating apoptosis. Other real-time measurements are shown in the SI, S3.4, Figure S12. *N* = 4–8 mice, 2–3 liver slices per mouse. Unpaired two-tailed Student’s *t* test was performed (**** indicates *p* ≤ 0.0001).

The basal OCR of two liver slices ranged between 6 and 8 pmol/min/μg (Figure 6A). According to the previous results (Figure 5B), this OCR correlates to viable liver slices, with an ATP content above 8 pmol/μg. After succinate treatment, we saw a rapid boost in mitochondrial OCR of 1.5 pmol/min/μg (Figure 6A). When succinate treatment was ceased, slice OCR did not return to its basal OCR (6 and 8 pmol/min/μg) but decreased drastically to 2 and 3 pmol/min/μg, respectively. This decrease was reversible, as the second treatment with succinate led to a similar value of OCR as the first succinate treatment (7.5 and 9.5 pmol/min/μg). When succinate treatment was again stopped, slice OCR decreased again to 2 and 3 pmol/min/μg. This decreased basal oxygen consumption was consistent with the ATP content measured in the same liver slices, which was extremely low, namely 1.0 pmol/μg (Figure 6B). The decreased basal OCR and reduced ATP content were also in agreement with the morphology, where the H&E staining showed necrosis and apoptosis after succinate treatment (Figure 6C, arrows). Thus, as confirmed by the ATP content and the number of viable cells scored with H&E staining, the severely decreased basal OCR that followed the substantially increased OCR due to succinate indicates that 40 mM succinate is toxic for the slice. We hypothesize that this concentration of succinate caused a depletion of the ATP precursor and a reverse transfer of electrons over the electron transport chain, resulting in a large number of reactive oxygen species rather than the production of ATP.^36,37^ This succinate-driven reverse electron transport has been reported at lower physiological concentrations of succinate that have accumulated in the cytosol during reperfusion/ischemia injury.^37^ Therefore, it is likely that the 40 mM succinate added to the incubation medium led to this reverse transfer of electrons in mitochondria, releasing a lot of reactive oxygen species and causing the observed cellular damage.

## Conclusions

For the study of complex (patho)physiological processes in the liver, a complex *ex vivo* model like the liver slice-on-a-chip model is highly suitable. To monitor (patho)physiological processes and tissue response to internal and external factors in real-time, oxygen sensing is essential.^9−11^ Oxygen sensing enables the determination of tissue viability and metabolic state during experiments. Additionally, real-time oxygen sensing provides precise control over the microenvironment of the tissue and information about tissue stability during experiments. Most studies of OOCs have made use of semiquantitative methods, such as foils or beads to measure oxygen consumption in the near exterior of cells.^9−11,18^ We have developed a microfluidic device with in-line integrated, optical oxygen sensors placed outside of the incubation chambers, for the detection of the total oxygen consumption of a liver slice in real-time. Real-time quantitative measurements of oxygen consumption of liver slices can be challenging because the fabrication of an oxygen-impermeable device that opens and closes is not always trivial. Bonding processes between two PC layers are difficult to optimize, and connecting the tubing in an oxygen-impermeable manner to the device is tricky. In our final device, we optimized the bonding process of the two PC layers and designed a holder for the chip to ensure proper closing of the third PC layer, the lid, and tubing connections.

By precisely measuring oxygen concentrations, we were able to successfully monitor the incubation conditions in our system and monitor the physiological health of tissues. To assess the physiological health of tissues with OCR, we correlated basal OCR with the well-known viability marker, ATP. We effectively monitored the oxygen consumption by liver slices and demonstrated a strong correlation between liver slice OCR and ATP content (*r* = 0.82, *p* < 0.0001). This correlation is unique, as such a strong correlation has not been reported previously. This correlation enables us to observe tissue viability during experiments without compromising tissue integrity. Thus, under controlled experimental conditions, basal OCR can be used as a proxy for ATP, facilitating the measurements of multiple parameters in a single tissue.

The total oxygen consumption of a cell consists mostly of mitochondrial oxygen consumption and is particularly relevant in the study of liver pathophysiology. With our system, we were able to demonstrate that we could monitor changes in mitochondrial OCR by actively modulating this parameter through periodic addition of the mitochondrial substrate, succinate. Oomen showed increased OCR with a single dose of succinate during an experiment.^19^ We have been able to develop this experiment further, boosting oxygen consumption sequentially with a couple of intermittent doses of succinate. This yielded the somewhat unexpected result that oxygen consumption could be boosted a second time by succinate addition, despite the detrimental effect of succinate on mitochondrial oxygen consumption after one dose. We also observed toxicity in liver slices, as was evident in basal OCR, ATP content, and tissue morphology. With these experiments, we show that our system is highly sensitive to changes in mitochondrial oxygen consumption and well-suited to monitor dynamic, oxygen-dependent processes in liver slices in real time. Our system offers an accessible approach for characterizing mitochondrial health in OOCs and can be easily implemented in future studies that utilize a microfluidic incubation setup. This is very relevant for human disease studies and drug discovery, as tissue response to therapeutic drugs can be measured in real time.

In conclusion, our incubation system for liver slices with integrated oxygen sensors promises to be an advantageous model for assessing liver (patho)physiology in response to potentially toxic drugs or lifestyle-related liver diseases. We have developed two generations of OOCs; while the first-generation device is suitable for small studies with limited resources and prototyping experiments, the second-generation device is designed for scalability and requires less expertise to produce reliable results. Future studies can make use of the two generations of devices developed here to investigate basal OCRs of human liver slices and examine how these rates respond to external factors.

## Supporting Information Available

Detailed description of the materials and methods; the system development and characterization of the first and second-generation devices; the viability assessment of the tissue in both devices and the other real-time measurements monitoring the effect of succinate treatment on slices, like the result shown in Figure 6 (PDF)
