# Supplementary material for: Real-Time Monitoring of Oxygen-Consumption Rate in Mouse Liver Slices Incubated in Organ-on-a-Chip Devices
Source: Anal Chem. 2024 Sep 30;96(40):15871–9. doi: 10.1021/acs.analchem.4c00355 (PMC11465219; doi:10.1021/acs.analchem.4c00355)
Supplement: Supplementary file 1 — ac4c00355_si_001.pdf [file ac4c00355_si_001.pdf]

Supporting information

Real-time monitoring of oxygen-consumption rate in mouse liver slices incubated in organ-on-a-chip devices

Ruby E.H. Karsten[†‡], Konstanze Gier[†], Jean-Paul S.H. Mulder[†], Maciej Grajewski[†], Peter Olinga[‡], and Elisabeth Verpoorte[†]*

† Pharmaceutical Analysis (XB20), Groningen Research Institute of Pharmacy, University of Groningen, 9713 AV Groningen, the Netherlands
‡ Pharmaceutical Technology and Biopharmacy, Groningen Research Institute of Pharmacy, University of Groningen, 9713 AV Groningen, the Netherlands

*e.m.j.verpoorte@rug.nl

Contents

S2. Materials & Methods 2
S2.1 Preparation and incubation of mouse precision-cut liver slices (PCLS) 2
S2.2 ATP and protein analysis 2
S2.3 Hematoxylin and Eosin (H&E) staining 2
S2.4 Technical drawings and description of chip features 2
S2.5 Reynolds number and Péclet calculations of oxygen and glucose 8
S2.6 Two-point oxygen sensor calibration 10
S2.7 Experimental design 10
S2.8 Derivation of the formula for the oxygen consumption of a slice 10
S3. Results 11
S3.1 System development and characterization 11
S3.2 Viability assessment of liver slices after incubation in the chip and well plate 13
S3.3 Basal OCR of liver slices 15
S3.4 Boosting the oxygen consumption of liver slices by succinate treatment 16
S4. References 17

S2. Materials & Methods

S2.1 Preparation and incubation of mouse precision-cut liver slices (PCLS)

Isofluorane/O_2 anesthesia was used to conduct surgical procedures on mice for liver excision. The excised liver was then placed in ice-cold UW medium (DuPont Critical Care, Waukegab, IL, USA). A biopsy punch was used to obtain 5 mm cores from the liver, and a Krumdieck Tissue Slicer (Alabama R&D, USA) filled with ice-cold saturated (95%O_2, 5%CO_2) Krebs Henseleit Buffer 1X (pH=7.4) was used to slice 250-μm-thick PCLS. The liver cores and PCLS were preserved in ice-cold UW medium during the slicing process. Freshly prepared PCLS were transferred to 12-well plates (Greiner bio-one GmbH, Austria) filled with 1.3 mL of Williams medium E (1X, glutaMAX-1, Gibco, UK), supplemented with 25 mM D-glucose monohydrate (Sigma Aldrich, UK) and 50 μg/mL gentamicin (Gibco, UK). PCLS were incubated (37°C, 80%O_2, 5%CO_2,15%N_2, shaking 90 times/minute) for 1 h or 24 h before transferring them to the microfluidic device or to freshly prepared well plates. Well-plate PCLS were collected at the same time as the PCLS for the microfluidic device and used as controls.

S2.2 ATP and protein analysis

After incubation, PCLS were individually snap-frozen in liquid nitrogen in 1 mL of sonication solution (70% ethanol and 2 mM ethylenediaminetetraacetic acid (EDTA), pH=10.9) and stored at -80°C until analysis. ATP was extracted by homogenization. Subsequently, the homogenate was centrifuged (16,000g at 4 °C for 5 min), and the pellet left to dry. The supernatant was collected separately. We used the ATP Bioluminescence assay KIT (Roche Diagnostics, Mannheim, Germany) to measure ATP content in the supernatant. The dried pellet was used to determine the total protein content of the PCLS using the Bio-Rad DC Protein Assay (Bio-Rad, Munich, Germany).

S2.3 Hematoxylin and Eosin (H&E) staining

Liver slices were dehydrated and cleared in xylene baths (Biosolve, the Netherlands), before being horizontally embedded in paraffin. Sections of 4-μm thickness were prepared with a microtome, then placed in a hot water bath (50°C), retrieved on a glass microscope slide (Klinipath, the Netherlands), and dried on a hot plate for 1 h (60˚C). Following deparaffinization in xylene and rehydration in graded ethanol baths, the sections were stained with hematoxylin (Klinipath, the Netherlands) and eosin (Sigma-Aldrich, the Netherlands). The sections were then left to dry, and a cover glass (VWR International, Belgium) was secured on top with DPX mountant (Sigma-Aldrich). The sections were imaged with a C96000 NanoZoomer (Hamamatsu Photonics, Hamamatsu, Japan) to obtain high-resolution digital images.

S2.4 Technical drawings and description of chip features

The chip structure consists of three incubation chambers with a common inlet and three separate outlets (Figures S1-S5). The first-generation microfluidic device consisted of three 1-mm-thick polycarbonate (PC) layers (3M, Bracknell, UK) (Figures S1, S2), while the second-generation device had three 4-mm-thick PC layers (Figure S3-S5). For both device designs, the structures in the individual parts were prepared by micromilling (Figures S1-S5). The lowest PC layer in each device contains the microfluidic channels and incubation chambers (Figures S1, S3). Channels had a width of 1.00 mm and depth of 0.25 mm, slice chambers had a diameter of 6.00 mm and depth of 0.25 mm (Figures S1, S3). The middle PC layer closes the microfluidic channels off, and contains four small cylindrical holes (first-generation device, d=0.95 mm, and second-generation device, d=1.30 mm) to serve as the microfluidic channel inlet and outlets (Figures S2, S4). The four through holes are centered in larger circular recesses, the dimensions of which are given (Figures S2, S4). This layer also embeds a row of three larger cylindrical holes (d=6.00 mm, h=1.00 or 4.00 mm, where h is equal to the thickness of the PC layer used to create accessible slice incubation chambers (Figures S2, S4).

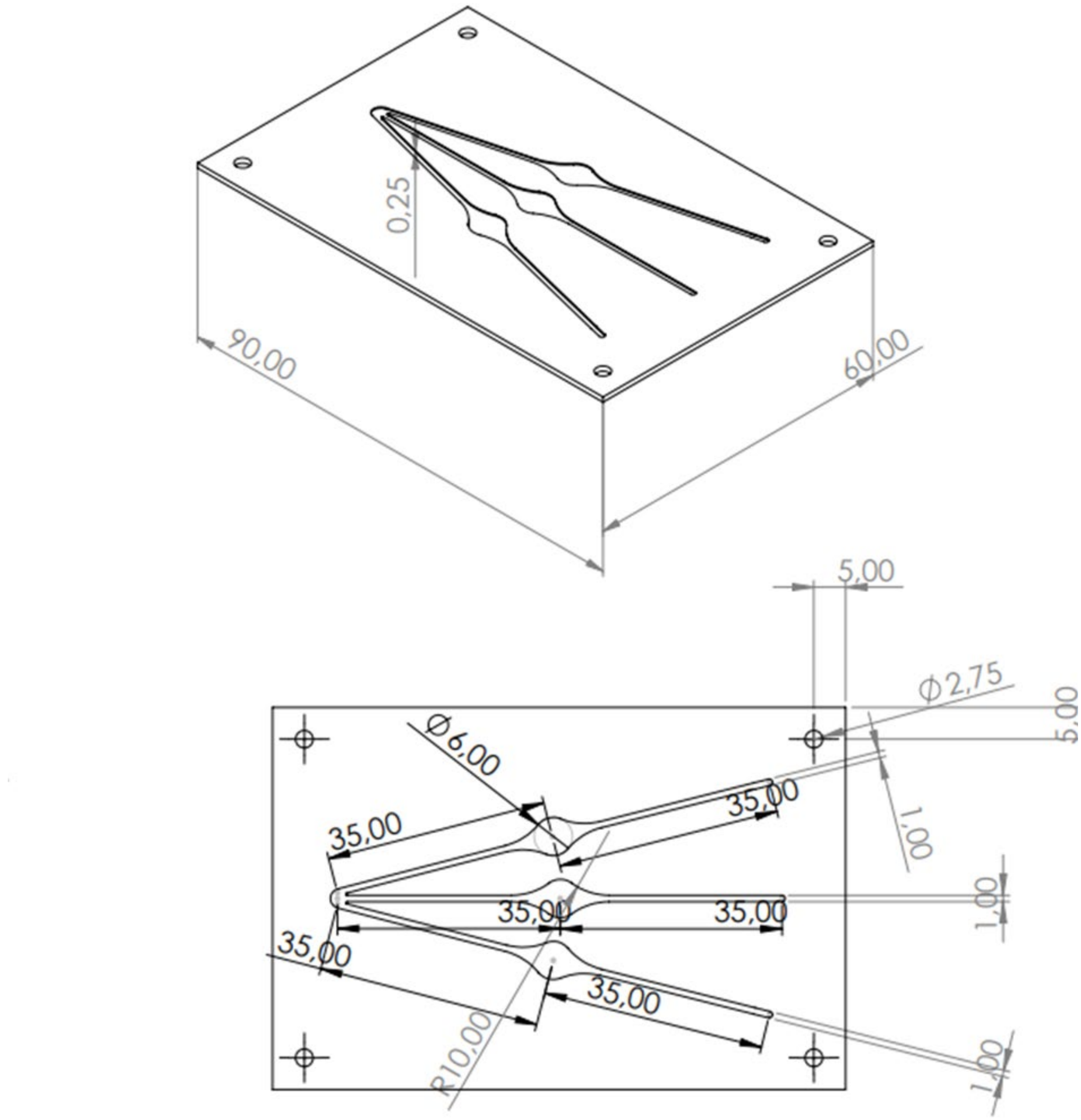

Figure S1 Technical drawing of the lower part of the first-generation microfluidic chip. The lower part contains the microfluidic channels and chambers for slice incubation. This chip is made of 1-mm-thick polycarbonate sheets. Drawings were made with Solidworks (2023 SP2.1).

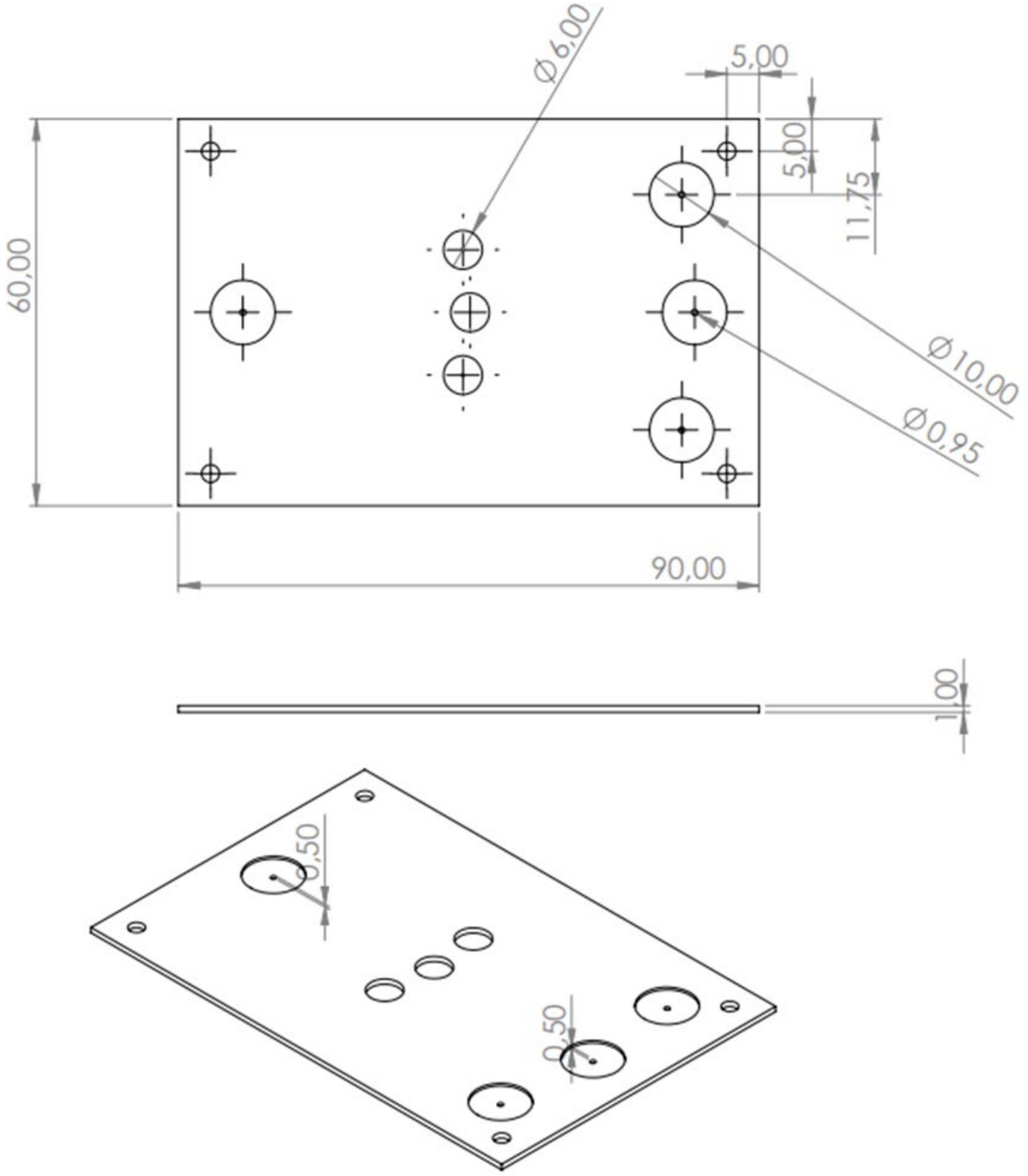

Figure S2 Technical drawing of the upper part of the first-generation microfluidic chip. This upper part closes the microfluidic channels, and has open structures for the inlet and outlets and liver slice incubation chambers (chamber volume 49.5 µL). This chip is made of 1-mm-thick polycarbonate sheets. Drawings were made with Solidworks (2023 SP2.1).

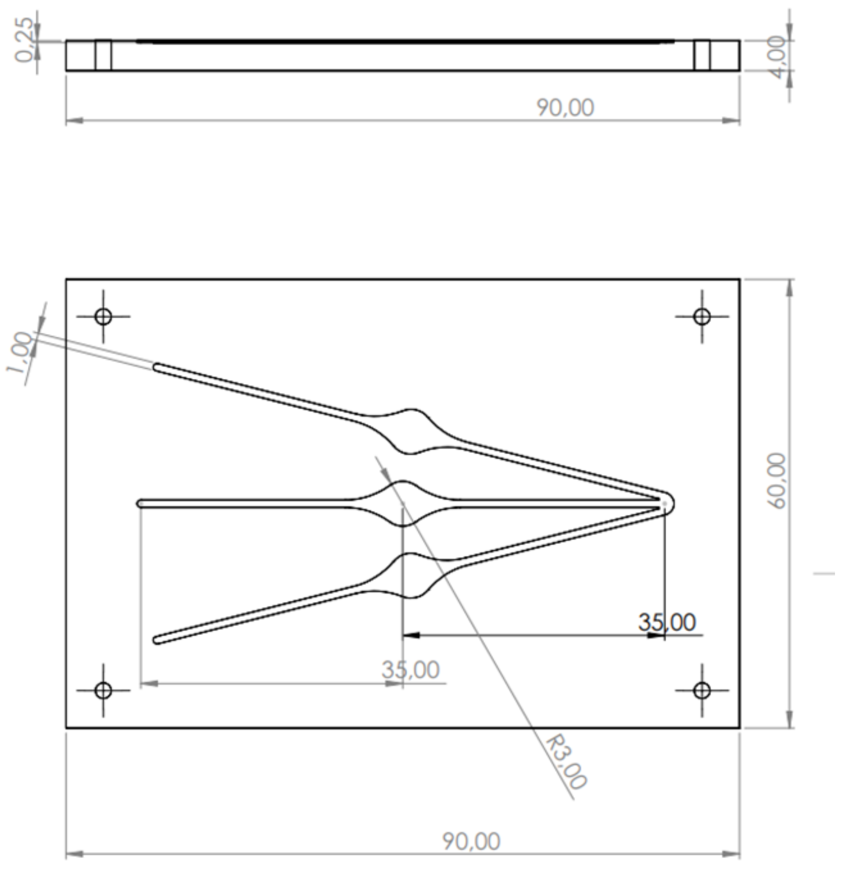

Figure S3 Technical drawing of the lower part of the second-generation microfluidic chip. The lower part contains the microfluidic channels and chambers for slice incubation. This chip is made of 4-mm-thick polycarbonate sheets. Drawings were made with Solidworks (2023 SP2.1).

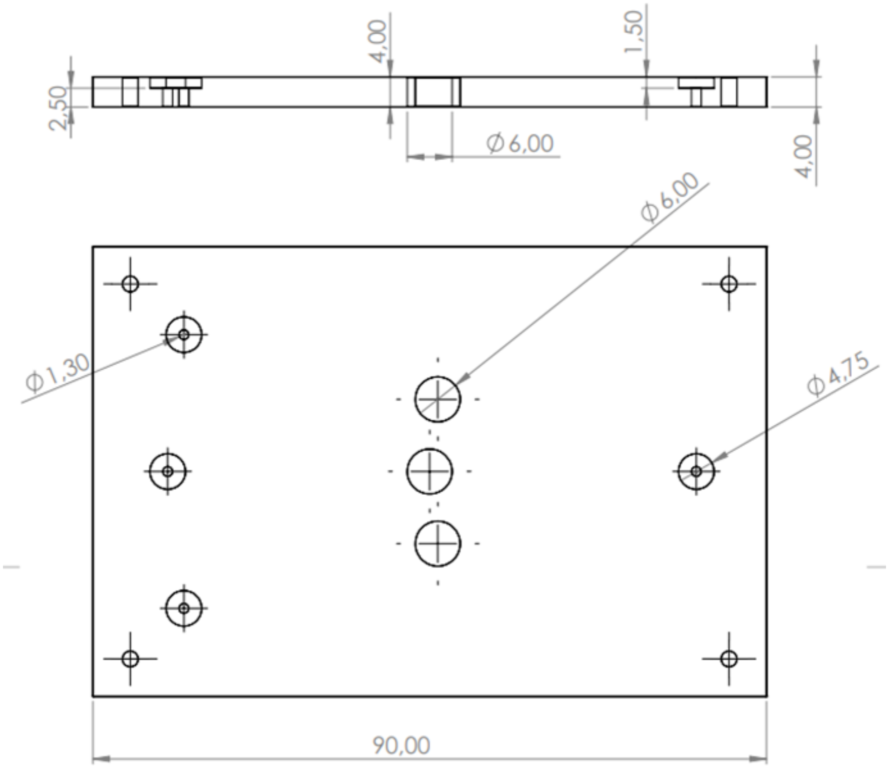

Figure S4 Technical drawing of the upper part of the second-generation microfluidic chip. This upper part closes the microfluidic channels, and has open structures for the inlet and outlets and liver slice incubation chambers. This chip is made of 4-mm-thick polycarbonate sheets. Drawings were made with Solidworks (2023 SP2.1).

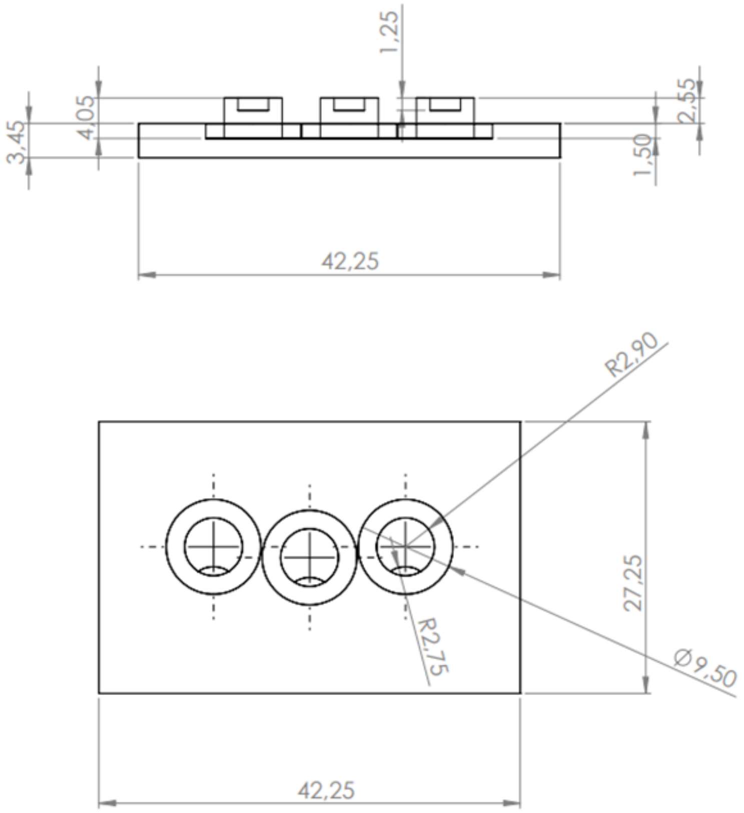

Figure S5 Technical drawing of the lid of the second-generation microfluidic chip. This part closes the liver slice incubation chamber reversibly and ensures a chamber volume of 49.5 µL. Drawings were made with Solidworks (2023 SP2.1).

The assembly of the three layers of the first-generation device is shown in Figure S6. The two lower PC layers of the first-generation device were bonded together with biocompatible adhesive tape (3M, Bracknell, UK). This device was then sealed to a third unstructured PC layer, the lid of the device, again using adhesive tape. To ensure that the incubation chambers were free of adhesive tape, three circles having the same cross-sectional dimensions as the chambers were cut out of the adhesive tape where the lid aligned with the chambers (Figure S6A). A new lid was used for every experiment. The assembled first-generation chip had a chamber depth of 1.70 mm (measured with a Gedore RED digital caliper (Conrad electronic SE, Hirschau, Germany)). 3D-printed Luer Lock connections were glued over the inlet and outlets of the chip with 2-component epoxy glue (Bison epoxy-glue, DA148B, Goes, the Netherlands). These 3D-printed Luer locks were then permanently connected by epoxy glue to four in-line integrated optical flow-through oxygen-sensors (FTCM- PSt7-02) (internal volume of 2.1 µL) (Presens, Regensburg, Germany). To ensure that there were no bubbles formed in the incubation chambers when sealing, the chambers were filled with medium, and drops of medium were added to the cut-out chamber ceilings on the lid. In the set-up of the first-generation device, the temperature was measured inside the incubator and manually entered into the software for calculating the oxygen measurements in the medium.

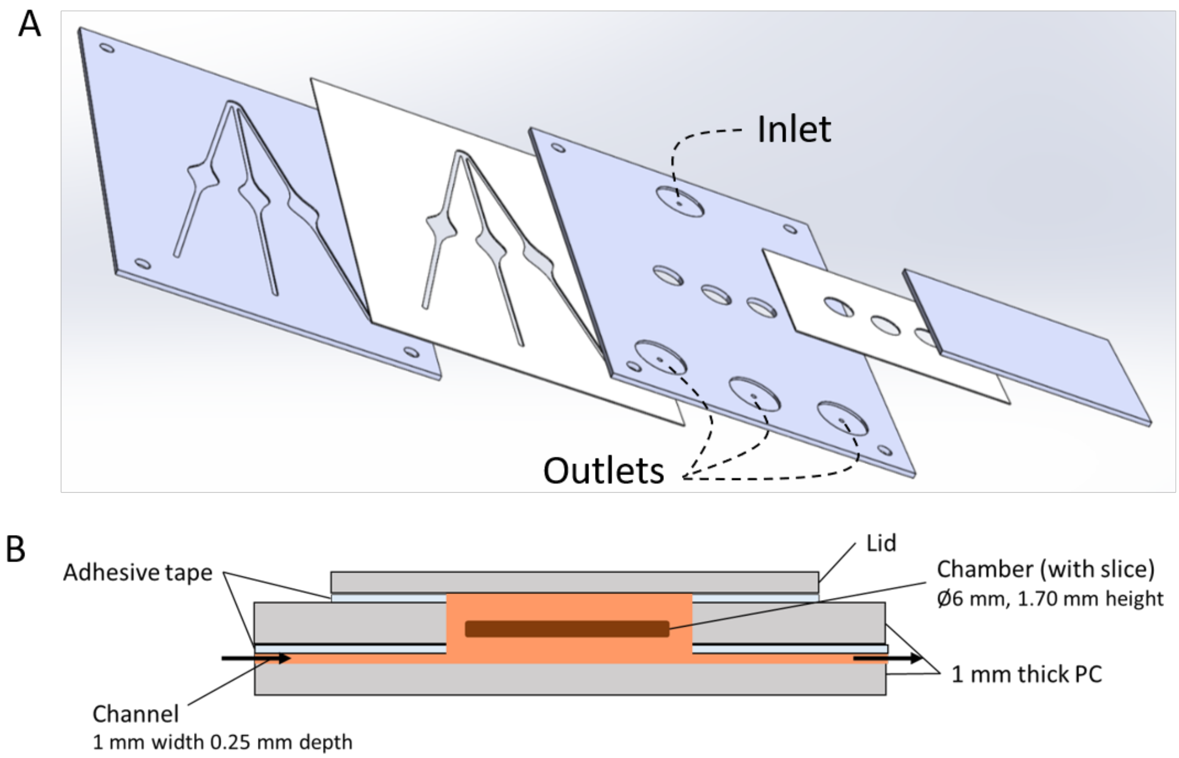

Figure S6. Assembly of the first-generation incubation chip. (A) Exploded view of the chip. Three 1-mm-thick polycarbonate (PC) pieces (grey) were prepared and assembled with double-sided tape (white). From left to right: Bottom PC part, adhesive tape, middle PC part, adhesive tape, and top PC part (lid). The bottom part contains the channels (1.00 mm width, 0.25 mm depth) and the chambers (Ø 6.00 mm, 0.25 mm depth). The middle part seals the channels and contains holes for the inlet (recess depth: 0.50 mm; diameter: Ø 10.00 mm; central hole diameter: Ø 0.95 mm), outlets (same dimensions as inlet), and incubation chambers (Ø 6 mm). The upper part (lid) closes the 48-µL incubation chambers. Lids were single-use; in other words, a new lid had to be made for each experiment. (B) Cross-section of the device containing medium (orange), and a liver slice (brown). Medium flow direction is indicated with arrows. The figure is not to scale.

S2.5 Reynolds number and Péclet number calculations of oxygen and glucose

Since the incubation chambers of both generations of the devices are irregularly shaped as ellipses, the Reynolds number (equation S3) and Péclet number (equation S4 and S5) vary throughout the channel. To give an idea of these dimensionless parameters in the space around the liver slice, we have calculated them at the widest point of an empty incubation chamber. The cross-section (perpendicular to the direction of flow) for both generations of devices at that point is a rectangle, with height of 1.7 mm and width of 6 mm.

Velocity of fluid (m/s)
Flow rate = 20 µL/min = 20 mm^3/min = 0.33.. mm^3/s

Cross-sectional area = $h{\cdot}w$ = 1.7 mm * 6.0 mm = 10.2 mm^2

h = height of rectangle (mm) = 1.7 mm

w= width of rectangle (mm) = 6.0 mm

$$V = \frac{flow\ rate}{cross\text{-}sectional\ area} = \frac{0.33..}{10.2} = 0.03267..mm/s$$

(S1) Formula for the velocity of fluid in a channel with rectangular cross-section (m/s)

Hydraulic diameter of a rectangle

$$\mathrm{D_h} = \frac{4(hw)}{(2h + 2w)} = \frac{4(1.7 \cdot 6.0)}{(2 \cdot 1.7 + 2 \cdot 6)} = 2.649..mm$$

(S2) Formula for the hydraulic diameter of the incubation chamber at its widest point

D_h = hydraulic diameter (mm)

h = height of rectangle (mm) = 1.7 mm

Reynolds number (Re) of water at 37 °C in a rectangle

$$Re = \frac{VD_\mathrm{h}\rho}{\mu} = \frac{0.032.. \cdot 2.6.. \cdot 0.9933}{0.6913} = 0.1244.. \approx 0.12$$

(S3) Formula for the Reynolds number (Re) of water at 37 °C in a channel having a rectangular cross-section

V = velocity of fluid (mm/s) = 0.03267..

ρ = density of fluid (mg/mm^3), water at 37 °C = 0.9933[1]

μ = dynamic viscosity of fluid (mg/mm·s), water at 37 °C = 0.6913[1]

D_h = hydraulic diameter (mm) = 2.649..

Péclet number for glucose in water at 37°C

$$Pe = \frac{VD_\mathrm{h}}{D} = \frac{0.032.. \cdot 2.6..}{0.000959} = 90.2816.. \approx 90.30$$

(S4) Calculation of Péclet number for glucose in water at 37°C

V = velocity of fluid (mm/s) = 0.03268..

D_h = hydraulic diameter (mm) = 2.649..

D = diffusion coefficient (mm^2/s), glucose in water at 37 °C = 0.000959 [2]

Péclet number for oxygen at 37°C in water

$$Pe = \frac{VD_\mathrm{h}}{D} = \frac{0.032.. \cdot 2.6..}{0.00324} = 26.7222.. \approx 26.72$$

(S5) Calculation of Péclet number for oxygen in water at 37°C

V = velocity of fluid (mm/s) = 0.03268..

D_h = hydraulic diameter (mm) = 2.649..

D = diffusion coefficient (mm^2/s), oxygen in water at 37 °C = 0.00324[3]

S2.6 Two-point oxygen sensor calibration

The first point was obtained in oxygen-free water, prepared by dissolving 1 g of sodium sulfite (Na_2SO_3) and 50 µL of cobalt nitrate ($Co(NO_3)_2$) in 100 mL of demineralized water. The 50 µL $Co(NO_3)_2$ was pipetted from a standard solution, which contained 1000 mg $Co(NO_3)_2$ /L in 0.5 M nitric acid. The second point was measured in 60% oxygen-saturated demineralized water (5% CO_2, 35% N_2). This way, the oxygen concentration in the medium could be accurately measured at all sensors, as the oxygen concentration varied from 80% at the inlet to about 40% at the outlets, depending on the oxygen consumption of the liver slices. Both points were measured at 37˚C and a flow rate of 20 µL/min.

S2.7 Experimental design

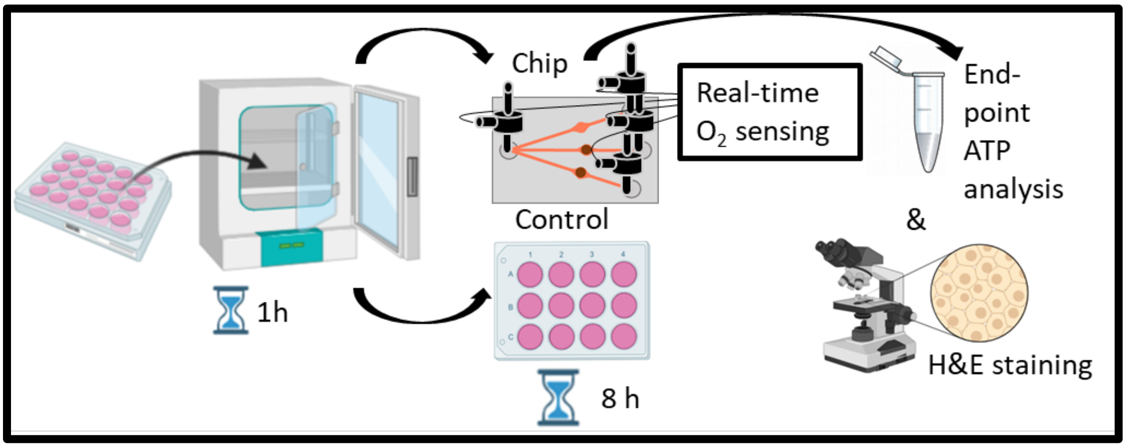

Figure S7 Schematic overview of the experimental design of the chip experiments. Before slices were incubated in the chip, they were incubated for at least 1 h in a well plate with Williams medium E supplemented with glucose and gentamycin (WMEGG), in an incubator set to 37˚C, and with a gas atmosphere of 80% O_2, 5% CO_2, 15% N_2. Hereafter, slices were incubated in the chip and the oxygen consumption rate (OCR) was measured in real time. Alongside the chip experiments, control slices were incubated in fresh WMEGG in a well plate. After incubation for 8 h, the slices were collected for either adenosine triphosphate (ATP) analysis, or hematoxylin and eosin staining (H&E) staining.

S2.8 Derivation of the formula for the oxygen consumption of a slice

The formula for the oxygen consumption of a slice is written in Equation (1) in the main text and was derived from:

(Coxy (pmol/µL) inlet – Coxy (pmol/µL) outlet, slice) – (Coxy (pmol/µL) inlet– Coxy (pmol/µL) outlet, empty) = Coxy (pmol/µL) consumed by liver slice.

(S6) The original formula for the oxygen consumption (pmol/µL) of liver slices during incubation. Coxy=oxygen concentration.

This formula can be rewritten to:

Coxy (pmol/µL) inlet – Coxy (pmol/µL) outlet, slice– Coxy (pmol/µL) inlet + Coxy (pmol/µL) outlet, empty = Coxy (pmol/µL) consumed by liver slice

(S7) The formula for the oxygen consumption (pmol/µL) of liver slices during incubation. Coxy=oxygen concentration.

Canceling out the Coxy inlet terms:

Coxy (pmol/µL) outlet, empty – Coxy (pmol/µL) outlet, slice = Coxy consumed by liver slice (pmol/µL)

(S8) The formula for the oxygen consumption (pmol/µL) of liver slices during incubation. Coxy=oxygen concentration.

Equation S8 was inserted in the following formula, Equation S9, calculating the final oxygen consumption rate (OCR) of a slice in pmol/min/µg protein:

$$OCR\ (pmol/min/\mu g) = \frac{(Coxy\ (pmol/\mu L)\ outlet\ \text{empty} - Coxy\ (pmol/\mu L)\ outlet\ slice\ )}{\mu g\ total\ protein\ of\ liver\ slice} \times (20\ \mu L/\text{min})$$

(S9) The formula for the oxygen consumption rate (OCR) (pmol/min/µg) of liver slices during incubation. Coxy=oxygen concentration. This is Equation (1) in the manuscript.

S3. Results

S3.1 System development and characterization

We determined the OCR of mouse liver slices in two generations of microfluidic devices using fiber-optic-based oxygen sensors placed at the inlet (one sensor) and each of the outlets of the three chambers (three sensors). In both device layouts, a common inlet provided medium to three separate slice chambers simultaneously. The geometry of the three channels containing chambers was identical, so that an initial flow rate of 60 µL/min was evenly split to 20 µL/min per chamber (See Materials and Methods *Chip design and fabrication* for a full description of the devices). The characterization of the second-generation device is shown in Figure S8. First, we calibrated the oxygen measurements by the sensors. Second, we determined the delay time of the system, meaning the time needed for the oxygen-enriched medium to reach the sensors at the inlet and outlets. The response time of the sensors can be neglected given that the oxygen sensors used have a response time of t90 < 5 seconds in liquids, as specified by the manufacturer. Third, we determined the time needed to reach equilibrium (steady signal) after a change in oxygen concentration. Note that the low-amplitude oscillation in Figure S8 is caused by the oxygen sensors continuously adjusting to the fluctuating temperature in the incubation box (Figure S8). These temperature fluctuations could be traced to the heater switching on automatically when the temperature dropped below the heater's set point of 37 °C. This oscillation at the inlet is at most equivalent to a concentration variation of 8.75 µmol/L starting at the time that the signal stabilizes, namely 1 hour after start of the experiment (Figure S8). This oscillation is not that large, given that the mean concentration is 787.7 µmol/L of oxygen; signal variation amounts just 1.1%. of the mean oxygen concentration. These variations should not affect the accuracy of the obtained oxygen consumption data, as the average values over a stable period were used in experiments. To minimize this oscillation, temperature sensors can be placed in more stable, contained environments.

The oxygen sensors were calibrated with a two-point calibration in the system at 0% oxygen (5% CO_2, 95% N_2) and 60% oxygen (5% CO_2, 35% N_2) to ensure minimum variation in signal within the expected concentration range. With the mass flow control system set at 60% oxygen, all sensors detected 60.3±0.7% in the medium, with an inter-experiment coefficient of variation (CV) of 1.1% (n=3 experiments) and an accuracy of 0.5%. This oxygen concentration is equivalent to 625 µmol/L (calculated with manufacturer software). When supplying the system nominally with 80% oxygen, the oxygen saturation detected in the liquid was 76.1±0.8% with an inter-experiment CV of 1.1%. The equivalent oxygen concentration is equal to 779 µmol/L (calculated with manufacturer software).

We then characterized the assembled system without slices present to determine the system's delay and equilibration times (Figure S8). The oxygen concentrations at the chip's inlet and outlets were monitored while oxygen-rich demineralized water was pumped through at a flow rate of 60 µL/min for the inlet and withdrawn at 20 µL/min from each outlet. During the characterization experiment, after the oxygen supply of the system was changed from 60% oxygen (5% CO_2, 35% N_2) (625 µmol/L) to 80% oxygen (5% CO_2, 15% N_2)(779 µmol/L), the time needed for the inlet oxygen sensor to show a change in oxygen concentration (delay time) was 0.97±0.02 minutes (n=3 experiments) (data from one experiment is shown in Figure S8). The delay time at the outlet sensors was 7.4±0.5 minutes (n=3 experiments). The equilibration time at the inlet, the time needed for the system to achieve a stable oxygen concentration was 17.7±0.6 minutes (n=3 experiments). The equilibration time at all three outlet

sensors was 53.0±1.7 minutes (n=3 experiments). This equilibration time corresponds to the equilibration time of the inlet multiplied by three, taking into account the flow rate reduction from 60 µL/min to 20 µL/min.

The whole system (4 sensors), at 80% oxygen, was fully equilibrated after 60.3±2.1 minutes (delay time + equilibration time) (n=3 experiments). First-generation devices had similar equilibration times as the second-generation device and were fully equilibrated after 58.0±3.0 minutes (3 experiments). The first-generation devices were characterized slightly differently from second-generation devices, with medium at 20% oxygen at the start followed by a switch to 80% oxygen (5% CO_2, 15% N_2) (Figure S9). Exact delay times and equilibration times of inlets and outlets in first-generation devices were not determined, but are expected to be the same, as the inner dimensions of the chip are the same in both generations.

There was one notable difference between the devices, without slices present, an oxygen gradient was observed in the first-generation device (Figure S9). The inlet oxygen concentration (80%) in the device was found to be higher than the oxygen concentrations at the outlets due to some loss of oxygen within the device itself (46%), due to imperfect sealing of the device. These outlet concentrations were the same in four independent experiments, indicating that oxygen loss was the same in all four cases (intra-assay variability between outlet sensors had a CV ranging from 0.35-2.87% at equilibrium, n=4 experiments). In second-generation devices, no oxygen gradient was observed because of improvements made to the device to prevent oxygen loss. Oxygen concentrations at the inlet and outlets were equal in the absence of slices (intra-assay variability between all sensors with a CV ranging from 0.10-1.61% at equilibrium, n=3 experiments). See Materials and Methods *Chip design and fabrication* for a description of the changes made to the device to minimize/eliminate oxygen loss. An exemplary graph of the oxygen measurements in the first-generation device during liver slice incubation is shown in Figure S10, and the second-generation device is shown in Figure 4 in the main text.

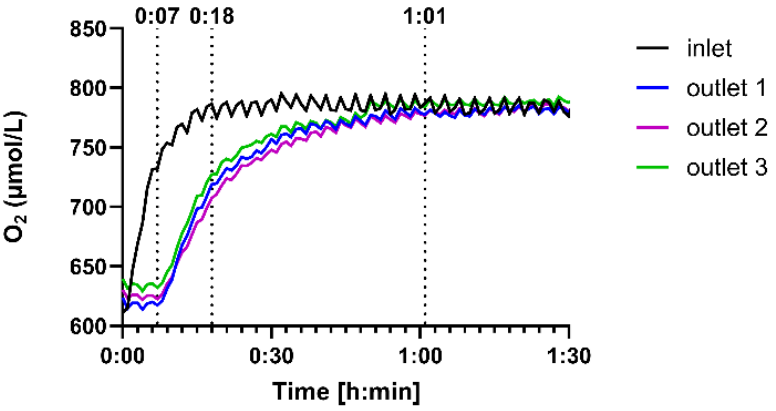

Figure S8 Example of an experiment run in the second-generation device using Williams medium E supplemented with glucose and gentamycin (WMEGG) at oxygen concentrations of 625 to 775 µmol/L at 37˚C for 1.5 h. Medium was equilibrated with 60% oxygen followed by 80% oxygen in the diffusion-based oxygenation box. The perfusion rate at the inlet is 60 µL/min, which is divided equally over three chambers, to yield perfusion rates of 20 µL/min each. The delay time of the inlet sensor to a change in oxygen concentration was 1 minute and of the outlets was 7 minutes. The inlet sensor was equilibrated in 18 minutes after a change in oxygen concentration, and the outlets after an equilibration time of 61 minutes.

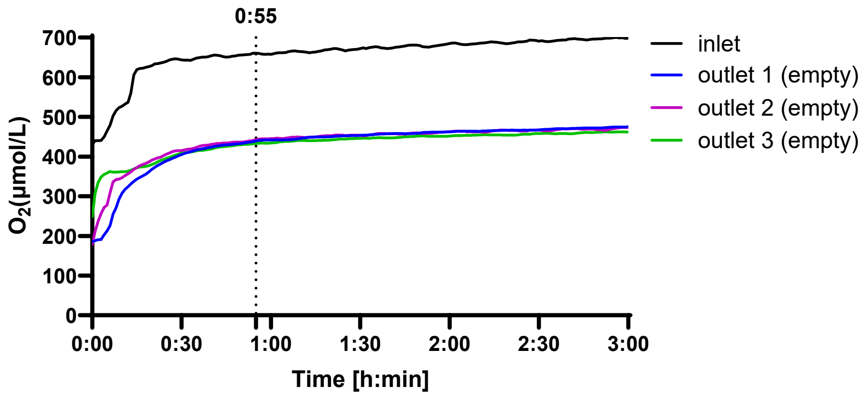

Figure S9 System run of the first-generation device with WMEGG at 37˚C for 3 h. The WMEGG was first saturated with 20% and then 80% oxygen in the diffusion-based oxygenation box. The perfusion rate at the inlet was 60 µL/min, which was then divided equally over three channels, each perfused at a rate of 20 µL/min. The medium in the system was equilibrated after 55 minutes.

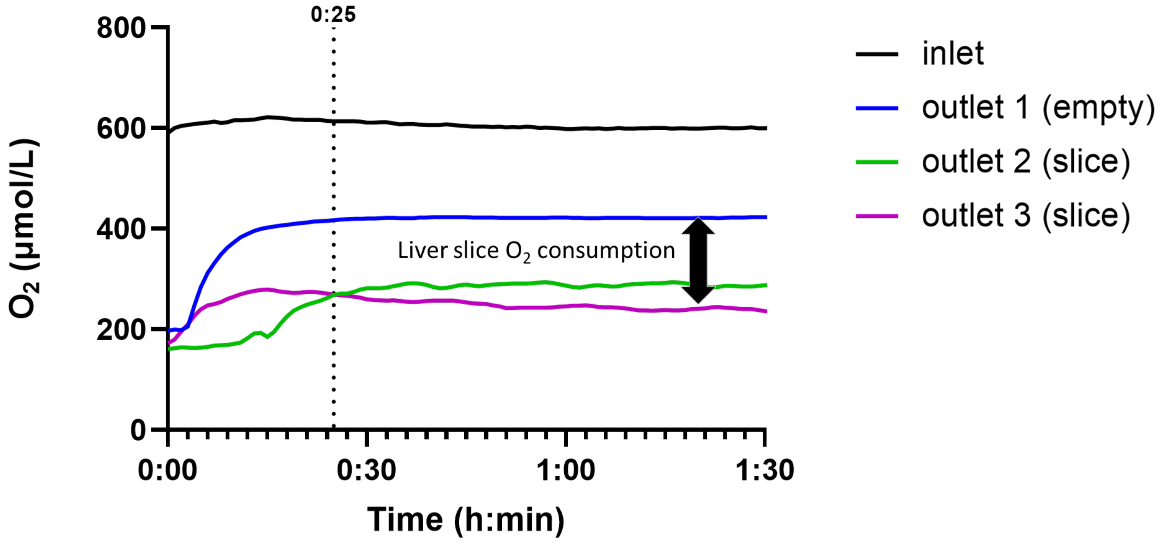

Figure S10 System run of the first-generation device saturated with WMEGG with 80% dissolved oxygen at 37˚C for 1.5 h. The WMEGG was saturated with 80% oxygen in the diffusion-based oxygenation box. The perfusion rate at the inlet is 60 µL/min, which was then divided equally over three channels, each perfused at a rate of 20 µL/min. Liver slices were incubated in chambers before outlet sensors 2 and 3. The chamber before outlet sensor 1 was empty and used as a reference to calculate the oxygen consumption of the liver slices (indicated with a black arrow). The medium flowing past outlet sensor 1 (empty) was equilibrated 25 minutes after introducing the liver slices and closing the device.

S3.2 Viability assessment of liver slices after incubation in the chip and well plate

To develop a functional liver-on-a-chip, we first investigated general liver slice viability after 8 h incubation in the chip under continuous flow, or in a well plate under agitation by shaking, by collecting the liver slices for endpoint morphology or ATP analysis (Figure S11). The H&E staining of the liver slices under flow showed 40-60% viable cells, which was the same as for liver slices in well plates (Figure S11A). The corresponding ATP content of liver slices incubated for 8 h under continuous flow was 4.7 pmol/µg, and under dynamic shaking was 7.9 pmol/µg protein (Figure S11B). The relation between this ATP level and tissue morphology has been previously described in the study of Westra *et al*, which showed that 5 pmol/µg ATP corresponds to about 50% viable hepatocytes[4]. Thus, the ATP content found under continuous flow is consistent with the viable hepatocytes (40-60%) scored with morphology. Yet, a significant decrease of 40.6% in ATP content was found for liver slices under continuous flow compared with those in the well plate (Figure S11B). The lower ATP content in liver

slices incubated under continuous flow is reproducible and furthermore consistent with previous work[5]. The reasons for differences in ATP content between slices incubated under flow and in well plates remain unclear. However, we showed that the viability, as measured by H&E staining, is very similar over 8 h in the chip versus well plates (Figure S11A). This result is consistent with a previous study that observed similar liver slice viability with H&E staining over 3 h of incubation[6]. Additionally, van Midwoud *et al* demonstrated that the viability, as measured by lactate dehydrogenase, alanine aminotransferase, and aspartate aminotransferase excretion, is very similar over 24 h in a chip versus well plate[7,8]. Furthermore, the slice function of the slice, such as its ability to metabolize the model compound 7-ethoxycoumarin was not affected in a chip over 3-24 h[6–8]. Although differences in ATP content remain unelucidated, indications are that both function and viability are no different than in standard well plates up to 8 h.

As our study aimed to demonstrate the feasibility of real-time OCR measurements with liver slices rather than to improve their viability, we concluded that the tissue viability of 40-60% viable hepatocytes was sufficient for our 8 h experiments. Therefore, we proceeded with this experimental design to investigate the OCR of liver slices. For future experiments with this device, it is recommended to conduct similar short-term studies or optimize the incubation conditions.

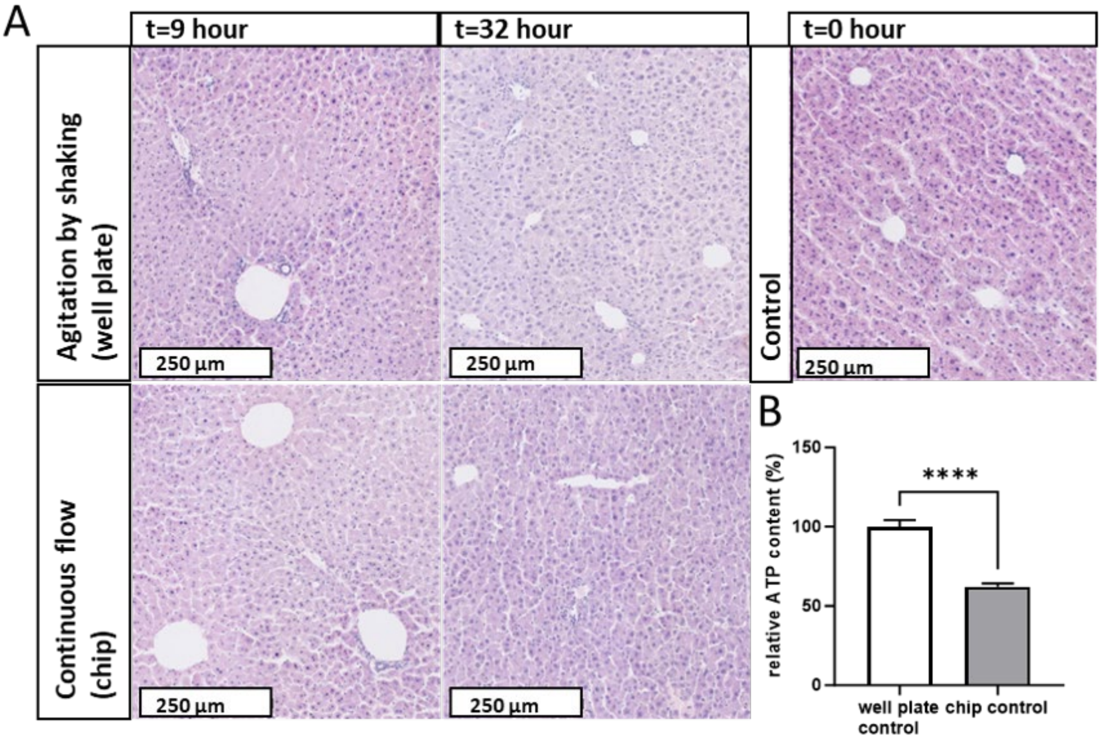

*Figure S11 Haematoxylin and Eosin (H&E) staining (A) showed that the viability of the tissue was unaffected by chip incubation, while intracellular ATP content (B) indicated a decrease in tissue viability compared to the well plate incubated liver slices. (A) H&E staining of liver slices incubated for 0 h, 1 h, or 24 h in well plates before 8 h of incubation in the chip or a freshly filled well plate control. Morphology scoring showed 60% viable hepatocytes and 40% necrosis for this mouse liver at 9 h and 32 h of incubation. (B) Relative ATP content in percentage to the well plate control ±SEM: of liver slices incubated for 1 or 24 h in well plates before 8 h of incubation in the chip or a freshly filled well plate. The flow rate was 20 µL/min. N=4 mice for (A) and 10 mice for (B), 1-3 liver slices per treatment per mouse. An unpaired two-tailed student's T-test was performed (*** indicates p≤0.001).*

S3.3 Basal OCR of liver slices

The individual data points for ATP content and OCR in Figure 5B, both before and after protein correction, are provided in Table S1.

Table S1 Adenosine triphosphate (ATP) content and oxygen consumption rate (OCR) of liver slices before and after correction for protein content of the slice. Data corrected for protein are used in Figure 5B. N=6 mice for the first-generation device, with 2-4 liver slices per mouse, and N=7 mice for the second-generation device, with 1 liver slice per mouse.

#Mice	OCR slice (pmol/min)	Protein slice (µg)	ATP slice (pmol)	OCR (pmol/min/µg protein)	ATP (pmol/µg protein)	#Device
M9	2206.56	645	1942	3.44	3.0	2nd generation
M8	2196.97	528	3341	4.17	6.3	2nd generation
M7	2577.85	882	2565	2.92	3.0	2nd generation
M6	2455.57	525	3527	4.68	6.7	1st generation
	2116.02	577	3200	3.67	5.5	1st generation
	1929.73	490	2170	3.94	4.4	1st generation
	2491.30	452	3380	5.52	7.5	1st generation
M5	3832.06	670	5258	5.72	7.9	1st generation
	2502.69	625	3059	4.00	4.9	1st generation
	2809.32	544	4208	5.17	7.7	1st generation
	2149.76	666	2795	3.23	4.2	1st generation
M4	2856.33	550	3775	5.19	6.9	1st generation
	2463.36	469	3047	5.25	6.5	1st generation
M3	2394.30	579	2090	4.14	3.6	1st generation
	2274.00	566	1962	4.02	3.5	1st generation
M2	2168.98	424	2307	5.12	5.4	1st generation
	2583.45	524	2611	4.93	5.0	1st generation
M1	2085.99	550	2000	3.79	3.6	1st generation
	3173.35	650	3225	4.88	5.0	1st generation
	2399.18	466	2661	5.15	5.7	1st generation
	2379.78	502	2516	4.74	5.0	1st generation

S3.4 Boosting the oxygen consumption of liver slices by succinate treatment

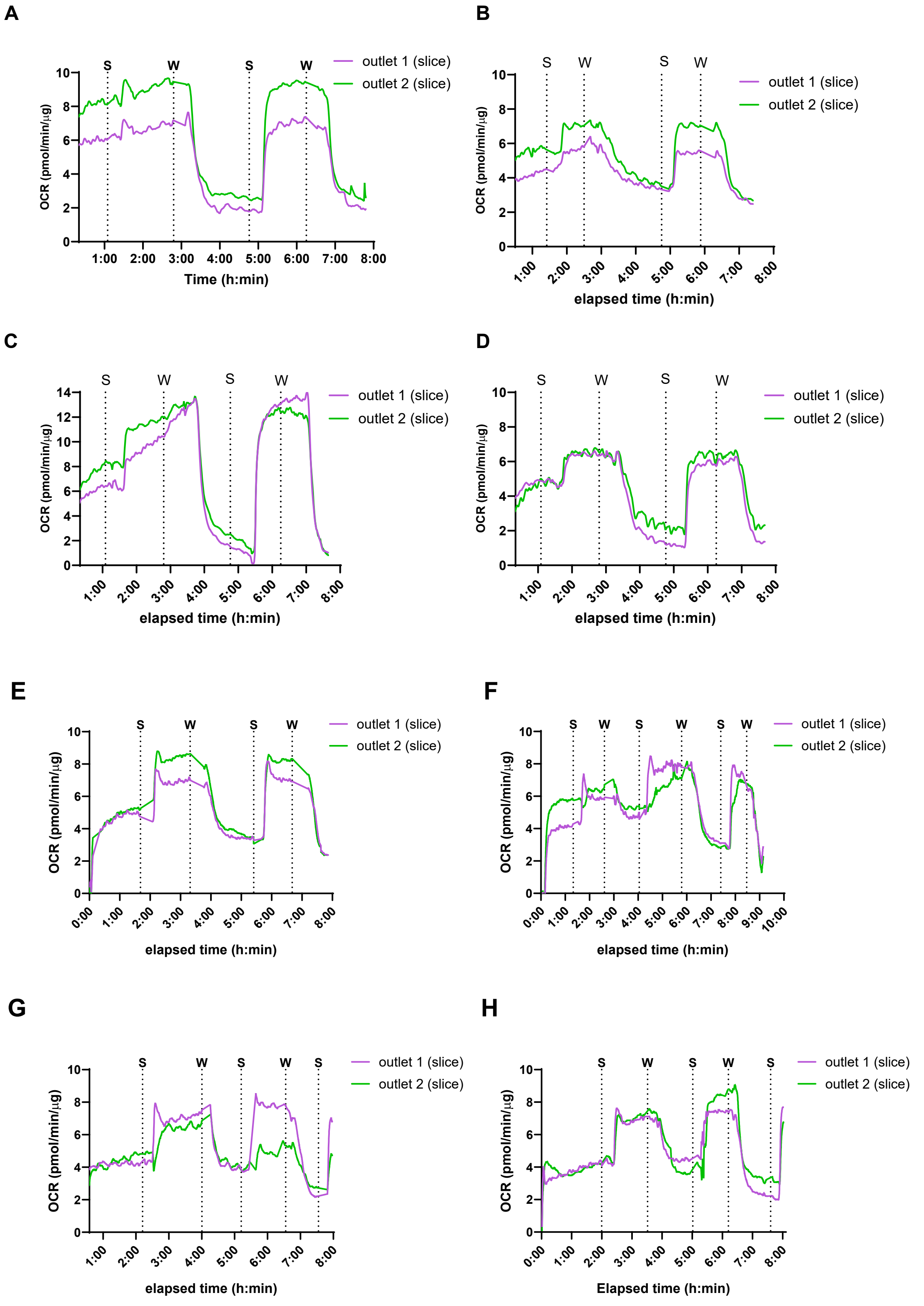

Figure S12 Rapid changes in oxygen consumption rate (OCR) of precision-cut liver slices (PCLS) in response to succinate treatment of 8 different experiments (A-H). Real-time OCR (pmol/min/µg protein) of two liver slices incubated for 1 h in a well plate followed by 8 h in the chip with Williams medium E supplemented with glucose and gentamycin (W) and treated twice with succinate (40 mM) (S). Succinate boosted mitochondrial oxygen consumption in liver slices twice to a maximum value. Removing succinate led to a drastic decrease in liver slice respiration, which was shown to be reversible by the second increase (n=8 mice, 2 slices/mouse).

S4. References

(1) Poon, C. Measuring the Density and Viscosity of Culture Media for Optimized Computational Fluid Dynamics Analysis of *in Vitro* Devices. *J. Mech. Behav. Biomed. Mater.* **2022**, *126*, 105024. https://doi.org/10.1016/j.jmbbm.2021.105024.

(2) Bashkatov, A. N.; Genina, E. A.; Sinichkin, Y. P.; Kochubey, V. I.; Lakodina, N. A.; Tuchin, V. V. Glucose and Mannitol Diffusion in Human Dura Mater. *Biophys. J.* **2003**, *85* (5), 3310–3318.

(3) Oomen, P. E.; Skolimowski, M. D.; Verpoorte, E. Implementing Oxygen Control in Chip-Based Cell and Tissue Culture Systems. *Lab. Chip* **2016**, *16* (18), 3394–3414. https://doi.org/10.1039/C6LC00772D.

(4) Westra, I. M.; Mutsaers, H. A. M.; Luangmonkong, T.; Hadi, M.; Oosterhuis, D.; de Jong, K. P.; Groothuis, G. M. M.; Olinga, P. Human Precision-Cut Liver Slices as a Model to Test Antifibrotic Drugs in the Early Onset of Liver Fibrosis. *Toxicol. Vitro Int. J. Publ. Assoc. BIBRA* **2016**, *35*, 77–85. https://doi.org/10.1016/j.tiv.2016.05.012.

(5) Oomen, P. E. Small Systems, Small Sensors: Integrating Sensing Technologies into Microfluidic and Organ-on-a-Chip Devices; [Thesis fully internal (DIV), University of Groningen]: University of Groningen, 2016; pp 77–97.

(6) van Midwoud, P. M.; Merema, M. T.; Verpoorte, E.; Groothuis, G. M. M. A Microfluidic Approach for in Vitro Assessment of Interorgan Interactions in Drug Metabolism Using Intestinal and Liver Slices. *Lab. Chip* **2010**, *10* (20), 2778–2786. https://doi.org/10.1039/c0lc00043d.

(7) van Midwoud, P. M.; Groothuis, G. M. M.; Merema, M. T.; Verpoorte, E. Microfluidic biochip for the perifusion of precision-cut rat liver slices for metabolism and toxicology studies. *Biotechnol. Bioeng.* **2010**, *105* (1), 184–194. https://doi.org/10.1002/bit.22516.

(8) van Midwoud, P. M.; Merema, M. T.; Verpoorte, E.; Groothuis, G. M. M. Microfluidics Enables Small-Scale Tissue-Based Drug Metabolism Studies with Scarce Human Tissue. *JALA J. Assoc. Lab. Autom.* **2011**, *16* (6), 468–476. https://doi.org/10.1016/j.jala.2011.07.003.
